# Identification of Mannose Interacting Residues Using Local Composition

**DOI:** 10.1371/journal.pone.0024039

**Published:** 2011-09-13

**Authors:** Sandhya Agarwal, Nitish Kumar Mishra, Harinder Singh, Gajendra P. S. Raghava

**Affiliations:** Institute of Microbial Technology, Chandigarh, India; University of Rome, Italy

## Abstract

**Background:**

Mannose binding proteins (MBPs) play a vital role in several biological functions such as defense mechanisms. These proteins bind to mannose on the surface of a wide range of pathogens and help in eliminating these pathogens from our body. Thus, it is important to identify mannose interacting residues (MIRs) in order to understand mechanism of recognition of pathogens by MBPs.

**Results:**

This paper describes modules developed for predicting MIRs in a protein. Support vector machine (SVM) based models have been developed on 120 mannose binding protein chains, where no two chains have more than 25% sequence similarity. SVM models were developed on two types of datasets: 1) main dataset consists of 1029 mannose interacting and 1029 non-interacting residues, 2) realistic dataset consists of 1029 mannose interacting and 10320 non-interacting residues. In this study, firstly, we developed standard modules using binary and PSSM profile of patterns and got maximum MCC around 0.32. Secondly, we developed SVM modules using composition profile of patterns and achieved maximum MCC around 0.74 with accuracy 86.64% on main dataset. Thirdly, we developed a model on a realistic dataset and achieved maximum MCC of 0.62 with accuracy 93.08%. Based on this study, a standalone program and web server have been developed for predicting mannose interacting residues in proteins (http://www.imtech.res.in/raghava/premier/).

**Conclusions:**

Compositional analysis of mannose interacting and non-interacting residues shows that certain types of residues are preferred in mannose interaction. It was also observed that residues around mannose interacting residues have a preference for certain types of residues. Composition of patterns/peptide/segment has been used for predicting MIRs and achieved reasonable high accuracy. It is possible that this novel strategy may be effective to predict other types of interacting residues. This study will be useful in annotating the function of protein as well as in understanding the role of mannose in the immune system.

## Introduction

Carbohydrates are important component of life, they are also known as third molecular chain of life, after DNA and proteins [1]. Protein-Carbohydrate interaction plays a vital role in a variety of biological processes like infection, immune response, cell differentiation and neuronal development [2]–[5]. In past large number of methods have been developed to predict protein-protein [6], protein-nucleotide [7], [8], protein-RNA [9], [10] and protein-DNA interaction [11]–[13]. Only limited number of methods has been developed to identify residues in proteins that interact with carbohydrate covalently (glycosylation) or non-covalently (carbohydrate binding sites) [14]–[21]. Most of the existing methods for predicting carbohydrate-binding sites are structure-based methods; these methods predict carbohydrate-binding sites in protein structures [15]–[19]. Thornton *et al*. (2000) predicted carbohydrate-binding site in known 3D protein-carbohydrate complex with overall accuracy of 65% [1]. Similarly, Balaji *et al*. (2004) developed a program COTRAN to predict galactose-binding site in known protein complexes and achieved 76% sensitivity [19]. Malik and Ahmad (2007) first time developed a sequence-based method for predicting carbohydrate-binding sites in a protein [20], [21]. Recently, classifiers have been developed for predicting glucose-binding sites in proteins [22].

**Figure 1:**
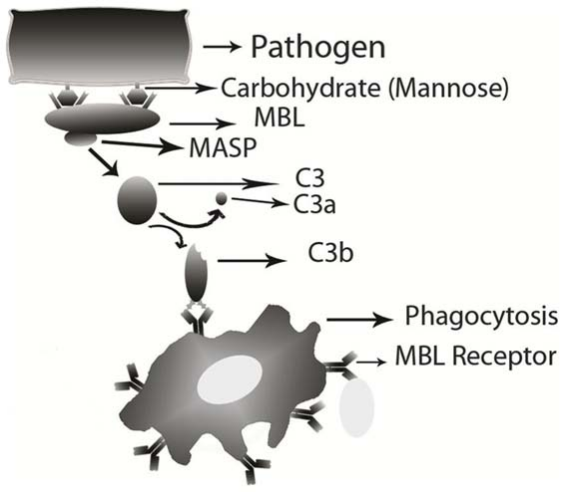
Schematic diagram of pathway of mannose binding lectins, recognition of mannose on pathogens and process of phagocytosis.

It is important to predict protein residues that interact with specific type of carbohydrate instead of any type of carbohydrate, in order to understand protein-carbohydrate interaction in depth. The goal of this study is to develop method for predicting mannose-interacting residues in a protein, a sugar monomer of the aldohexose series of carbohydrates [23], [24]. The mannose binding proteins (MBPs) also called mannose-binding lectin (MBL) (24), plays a vital role in immune defense mechanism. These MBL mediates innate immune function including activation of lectin complement pathway, by binding to mannose on the surface of wide range of pathogens that are absent at mammalian cell surface [3]. These mannose binding proteins play an important role in opsonize bacteria by tagging the surface of a pathogen to facilitate recognition and ingestion by phagocytes (Figure 1). Opsonization is a process to make bacteria or other cells more susceptible to the action of phagocytes [2], [3].

In the present work a systematic attempt has been made to develop modules for predicting mannose interacting residues in a protein, from its primary sequence. Firstly, we developed similarity-based module for predicting MIRs in proteins [25]. Secondly, we developed Support Vector Machine (SVM) based modules for predicting MIRs in proteins using binary profile of patterns [26]. Thirdly, SVM module was developed using evolutionary information in form of PSSM profile [27], [28]. Finally, a module based on local composition or composition profile of patterns was developed for predicting MIRs in proteins.

## Materials and Methods

### Dataset

**Figure 2:**
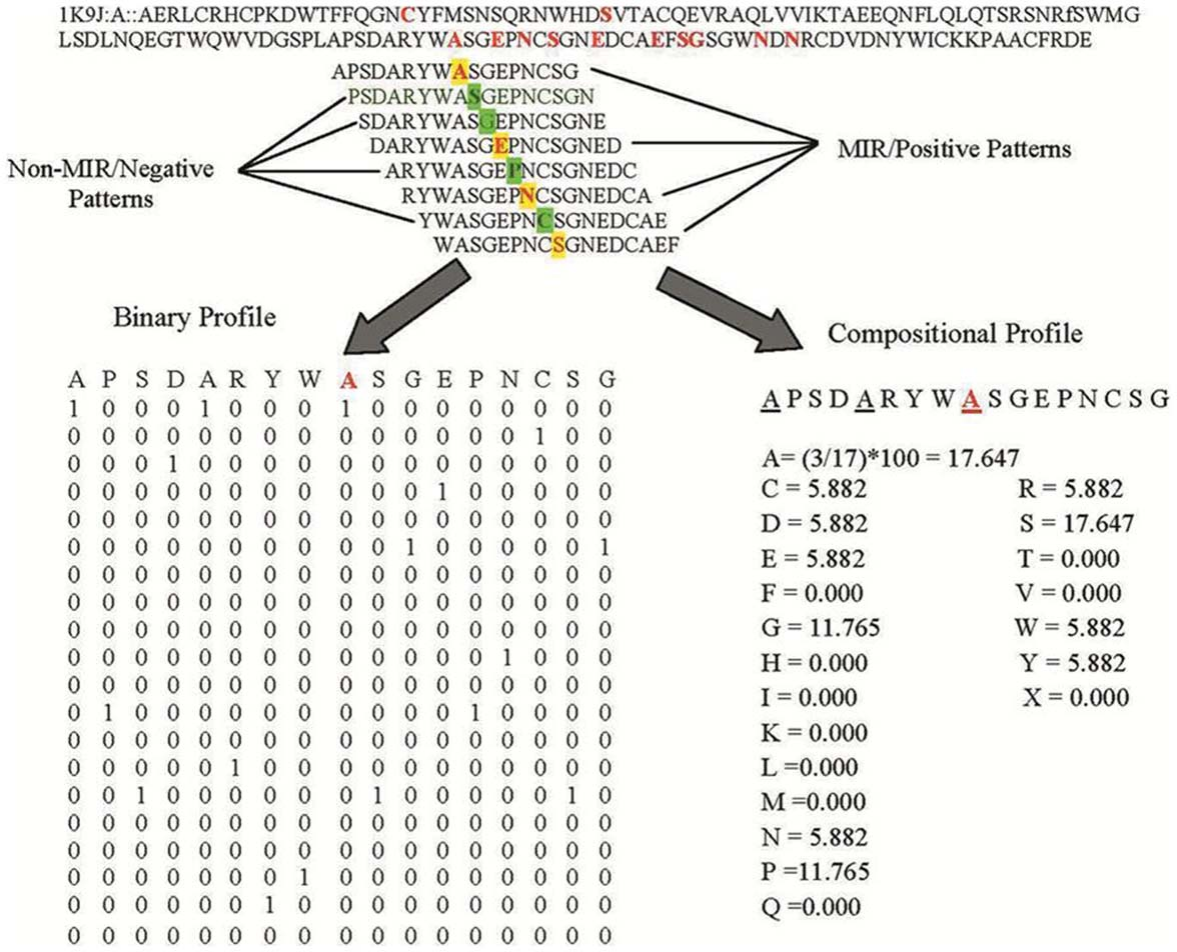
Schematic representation of algorithm used to generate patterns from a given protein sequences and their binary and composition profile.

We extracted 647 structures of mannose-binding proteins from Protein Databank (PDB). These mannose-binding proteins were selected based on information provided in SuperSite documentation [29]. The chains of these proteins were processed using Ligand Protein Contact (LPC) server [30] and got total 1502 PDB chain which contain mannose-interacting residues. Figure 2, shows a mannose protein complex with their MIRs. Further Blast-clust (http://blast.ncbi.nlm.nih.gov/Blast.cgi) software was used for removing redundant chains. Finally we got 120 mannose binding protein chains, where no two chains have more than 25% sequence similarity. These chains contain 1029 mannose-interacting and 38136 mannose non-interacting residues (binding sites). Mannose binding site is defined as the site present on the surface of protein, where mannose atoms interact with the amino acids of protein within a distance-cutoff of 4 A°. Sequences of these 120 mannose-binding proteins with their PDB ID and chain name are available at http://www.imtech.res.in/raghava/premier/data.php, where MIRs are in lowercase and non-MIRs are in uppercase.

### Creation of Patterns

It is well known that the function of a residue is not solely determined by itself but influenced by its neighboring residues [7], [8]. Thus, we created overlapping patterns (segments) of different window size from 17 to 25 residues for each mannose-binding protein. If the central residue of pattern was MIR, then we classified the pattern as positive (or mannose interacting) pattern otherwise it was termed as negative (or non-interacting) pattern. To create a pattern corresponding to the terminal residues in a protein chain, we added (L-1)/2 dummy residues “X” at both terminals of protein (where L is the length of pattern) [9]. It means for window size 17, we added 8 “X” at N terminal and 8 “X” at C-terminal, in order to create L patterns from sequence of length L. It is similar to the approach adopted by Kaur and Raghava [27]–[28] for prediction of turns in protein sequences (Figure 2).

### Main Dataset

In this dataset we have used equal number of positive and negative patterns, where negative patterns were randomly picked up from the pool of negative patterns. Positive patterns contain interacting residues in its center while negative patterns contain non-interacting residues in its center. We have used this dataset because machine-learning techniques are more efficient in learning when negative and positives patterns are equal and it's common in literature. In summary main dataset consists of 1029 interacting and 1029 non-interacting patterns.

### Realistic Dataset

Though it's easy to develop the model on equal dataset but it does not represent the realistic situation. In real life non-MIRs are much more than MIRs. This raises question whether models developed on our main dataset will be effective in real life. To overcome this problem we created a realistic dataset, which contain more non-interacting patterns then interacting patterns. This dataset has 1029 MIR Patterns and 10320 non-MIR patterns (approximately 10 times more negative pattern of the positive patterns). In this dataset we used only 10320 non-MIRs out of total 38136 non-MIRS in order to save computational time used to train/test SVM models.

### Binary Profile of Patterns

We created positive and negative patterns as described above but these patterns cannot be used directly for developing SVM based models because SVM need numerical values. Thus we converted these patterns into binary numbers, where a pattern of length N was represented by a vector of dimension N × 21. Each amino acid is represented by a vector of dimension 21 (e.g. Ala by 1,0,0,0,0,0,0,0,0,0,0,0,0,0,0,0,0,0,0,0), contained 20 amino acids and one dummy amino acid X (Figure 2). This binary profile of patterns has been used in most of existing methods [7]–[12].

### Evolutionary Information

volutionary information of protein sequences were obtained from position-specific scoring matrix (PSSM) generated using PSI-BLAST [18], where each mannose-binding protein was searched against non-redundant (nr) database (ftp://ftp.ncbi.nih.gov/blast/db/fasta/nr.gz) of protein sequences. The PSSM matrices were generated by PSI-BLAST using three iterations at cutoff e-value of 0.001. The PSSM thus generated contained the probability of occurrence of each type of amino acid residues at each position along with insertion/deletion. PSSM profile encapsulates evolutionary information in the form of a matrix, which is considered as a measure of residue conservation at a given location. This means that evolutionary information for each amino acid is encapsulated in a vector of dimension 21, where the size of PSSM matrix of a protein with N residues is 21×N. Where 20 dimension are standard amino acid and 1 for dummy amino acid. We normalized each value within 0–1 ranges using following equation:
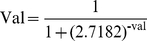
(1)Where *val* is the PSSM score and *Val* is its normalized value. We normalize values of PSSM matrix, as variation was very high between –1000 to +1000. It is difficult for SVM to learn from these types of variation, thus we normalize values between 0 and 1.

### Local Composition or Composition Profile of Patterns

In previous studies, patterns or segment were converted into binary numbers, where a vector of dimension 21 represents an amino acid. In this study we used local composition or composition profile of patterns (CPP). It means we represent a pattern by its amino acid composition. Thus a vector of dimensions 21 can represent a pattern or segment of any length. Recently, our group used this concept for predicting conformational B-cell epitopes [31]. In CPP, we simply compute amino acid composition of a pattern (Figure 2). Thus pattern can be represented by a vector of dimension 21, which represents twenty natural amino acids and one dummy amino acid “X”. Amino acid composition of patterns were computed using following formula [31], [32]:
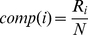
(2)where ***comp(i)*** is the fraction of residue or composition of residue of type ***i***. ***Ri*** and ***N*** are number of residues of type ***i,*** and total the number of residue in protein ***i*** (length of protein) respectively.

### Support Vector Machine (SVM)

SVM based modules have been developed for discriminating MIRs and non-MIRs in proteins [26]. SVM is a universal approximator based on statistical learning and optimization theory, which support both regression and classification. SVM is particularly attractive to biological sequence analysis due to its ability to handle noise, dataset and large input space. We implemented SVM technique using SVM_light package (http://www.cs.cornell.edu/People/tj/svm_light) [26]. This package is very powerful and users friendly, which allow users to select various parameters and various kernel functions, like radial basis function (RBF), linear and polynomial functions.

### Five-Fold Cross-Validation

In this study, we used commonly used technique called five-fold cross-validation technique, were data set is randomly divided into five subsets, each containing an equal number of patterns [6]–[8]. Each set is an unbalanced set that retains nearly equal number of interacting and non-interacting patterns. Out of these five sets four sets were used for training and the remaining fifth set for testing. This process was repeated five times in such a way that each set was used once for testing. The final performance was obtained by averaging the performance of all the five sets.

### Performance Measures

In order to assess the performance of SVM modules developed in this study, we used standard parameters [6]–[13]. These parameters have been described in brief in this section, for detail description see Chauhan *et al*. [8]. We compute following threshold dependent parameters; i) sensitivity is percent of correctly predicted MIRs, ii) specificity (Spe) is percent of correctly predicted non-MIRs, iii) accuracy (Acc) is percent of correct predicted residues and iv) Matthew's correlation coefficient (MCC). In this study we also evaluate our models using area under curve (AUC), which is a threshold independent parameter. We used SPSS package (11.0.1) for plotting ROC curve and for calculating AUC (http://www.spss.com/).

## Results

### Analysis of MIRs

**Figure 3:**
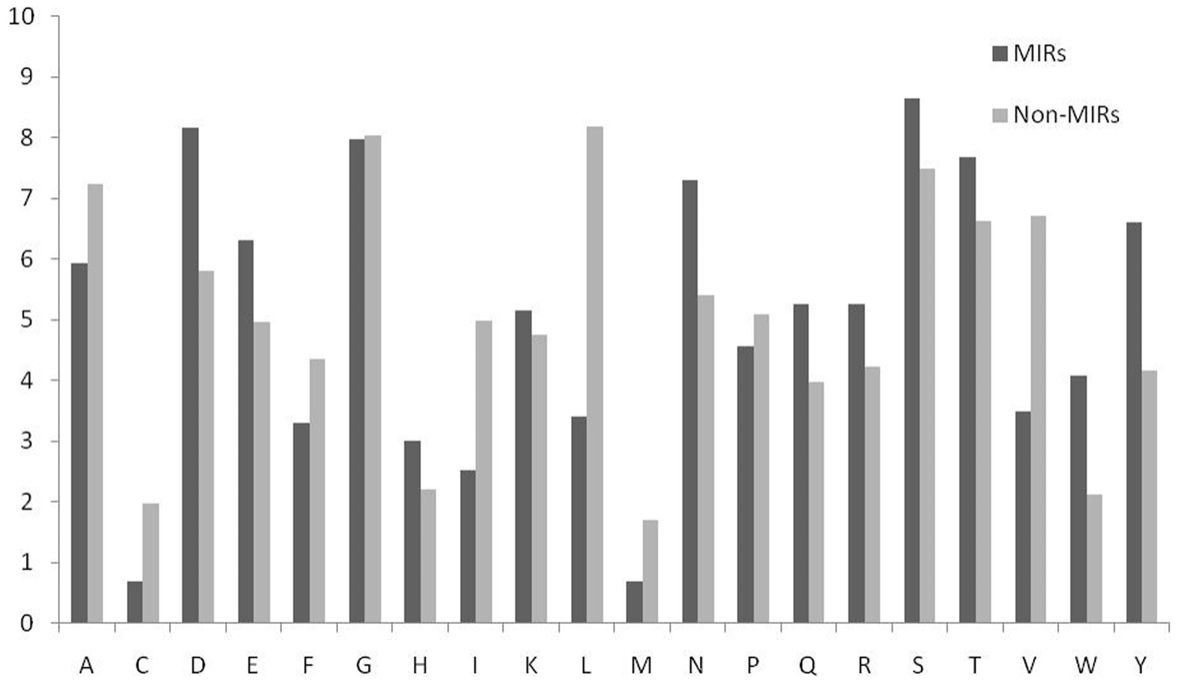
Comparison of percent amino acid composition of mannose interacting and non-interacting residues.

**Figure 4:**
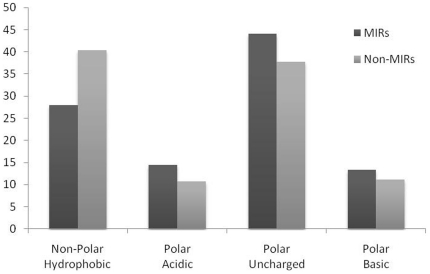
Comparison of percent composition of MIRs and Non-MIRs based on properties of residues.

**Figure 5:**
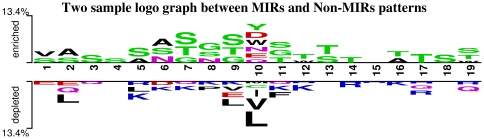
Two Sample logo graph between MIRs and Non-MIRs patterns.

In order to understand whether certain types of residues are preferred in mannose interaction, composition of interacting and non-interacting residues was compared (Figure 3). It was observed that certain types of residues like Asp, Glu, Asn, Gln, Arg, Ser, Thr, Trp and Tyr are preferred in mannose interaction (Figure 3). Majority of the amino acid that helps in protein carbohydrate interaction are the one having side chains residues with polar groups like ASN, ASP, GLU, GLN, ARG and HIS [33]. Amino acid side chains of tryptophan and tyrosine are capable of making CH/pi interactions with carbohydrates. In CH/pi interactions the hydrophobic C-H groups of carbohydrate interact with the pi-electron system of aromatic-acid residues. These CH/pi interactions are important for carbohydrate binding proteins for ligand-recognition [34]. We have also observed that polar/uncharged amino acids play an active role to differentiate between MIRs and Non-MIRs (Figure 4). The dominance of these residues shows a vital role of these residues in mannose interaction. It has been shown in the past that properties of a residue (i.e. interaction, secondary structure) depend on its neighbor residues [7]. It is a common practice to develop a method using window/pattern where center residue is interacting and non-interacting [8]–[12]. For better understanding, we create a two-sample logo graph showing MIR at center is different than non-MIR (Figure 5). From the Figure 4 we found that Asp, Tyr, Trp, Asn, Glu and Gln residues are abundant in center position and flanked by mostly Ser, Thr, and Gly in positive patterns/MIRs Patterns.

**Figure 6:**
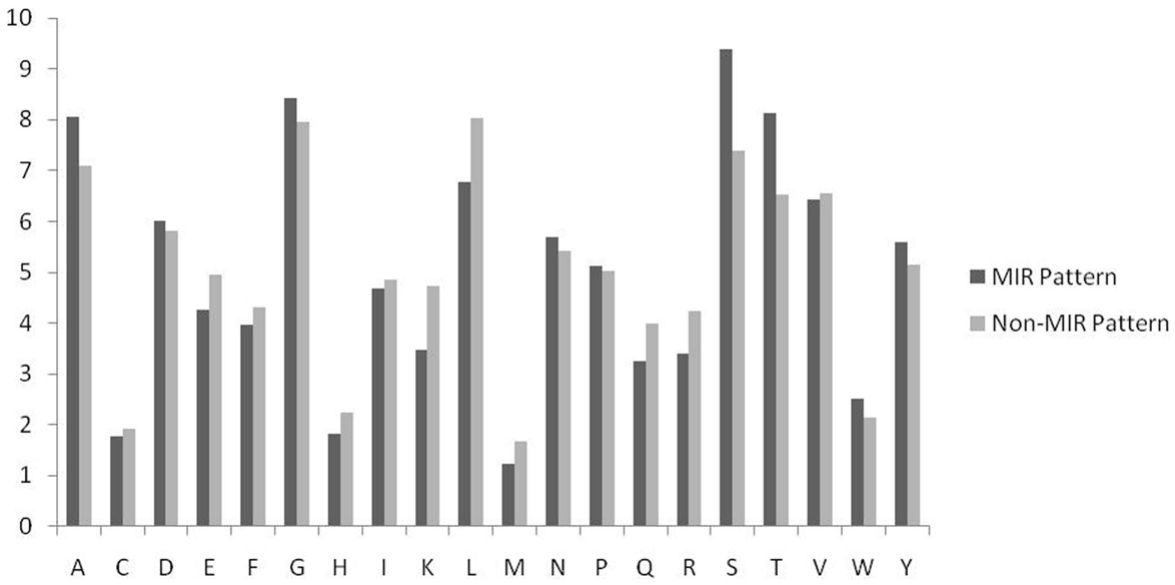
Overall amino acids comparison of MIRs and Non-MIRs patterns.

In addition we have also created a graph for comparing composition of MIRs and non-MIRs containing patterns as shown in Figure 6. It was seen that Ser and Thr residues are prominent in MIRs patterns. It was interesting observation compared with other analysis, such as DNA and RNA binding proteins, which have high preference for charged residues at the binding sites, transmembrane helical proteins with a stretch of hydrophobic residues in the membrane [35]. Here, Arg is favored and Lys is not favored; among aromatic residues, Tyr and Trp are favored and Phe is not favored.

### Similarity Based Module

BLAST is a commonly used tool for annotating function of a protein [25]. In this technique protein is searched against database of annotated proteins (e.g., Swiss-Prot). If a query protein or its region has high similarity with an annotated protein then we assign same function to a query protein. BLAST was examined whether it can be used for predicting mannose-interacting residues in proteins. Mannose-binding proteins (MBP) were searched against remaining MBPs (119 MBPs), this process is repeated 120 times in such a way that each MBP was searched against remaining MBPs. It was observed that we got BLAST hit only for 40 MBPs, among those we analyzed 12 MBPs, which have minimum E-value and has more than three mannose interacting residues. Alignment details (BLAST) of each protein are shown in Datasheet S1. It was observed that BLAST was not suitable for predicting MIRs. There is a need to develop an alternative technique for predicting MIRs.

### Performance of SVM on Main Dataset

#### Binary Profile of Patterns

It has been shown in previous studies that 17-residue patterns gave optimize performance in prediction of nucleotide interacting residues [8]–[12]. Thus we also developed SVM based model using patterns where length of a pattern is 17-residues. These patterns were converted into binary patterns called binary profile of patterns. We achieved maximum MCC of 0.19 with accuracy 59.60% using binary profile of patterns of length 17 (Table 1). At zero threshold accuracy was maximum and having minimum difference in sensitivity and specificity. Model was evaluated on main dataset using fivefold cross-validation technique. Although this is a standard technique for predicting interacting residues, unfortunately the performance of this technique was very poor in case of MIR prediction.

**Table 1:** The performance of SVM models developed on main dataset (Window length 17) using binary, evolutionary and compositional profile (complete table shown in Table S1).

Binary					PSSM				Composition			
Thes	Sen	Spe	Acc	MCC	Sen	Spe	Acc	MCC	Sen	Spe	Acc	MCC
−0.3	81.21	30.34	55.77	0.13	85.6	36.76	61.18	0.26	96.77	41.64	69.21	0.46
−0.2	73.44	39.43	56.44	0.14	82.58	43.91	63.24	0.29	93.84	48.39	71.11	0.47
−0.1	66.09	49.44	57.76	0.16	78.05	50.15	64.1	0.29	87	66.47	76.74	0.55
**0**	**58.43**	**60.78**	**59.6**	**0.19** *	73.51	57.8	65.66	0.32	**77.03**	**82.89**	**79.96**	**0.60**
0.1	49.74	68.54	59.14	0.19	**67.37**	**63.54**	**65.46**	**0.31**	68.82	90.13	79.47	0.60
0.2	40.65	75.89	58.27	0.18	59.01	68.68	63.85	0.28	63.44	94.62	79.03	0.61
0.3	31.56	82.89	57.2	0.17	51.56	73.82	62.69	0.26	56.79	96.48	76.64	0.58

*Bold values indicate the point where sensitivity and specificity is equal or minimum difference with maximum MCC.

### SVM Model Using Evolutionary Information

In the past, it has been shown in several studies that evolutionary information provides more information then single sequence [27], [28]. In this study, the evolutionary information obtained from a PSSM profile generated using PSI-BLAST has been used for developing SVM based models[25]. As shown in Table 1, performance increased significantly when PSSM was used as input instead of single sequence (Table S1). We achieved maximum MCC of 0.32 with accuracy 65.66%, sensitivity 73.51% and specificity 57.80%.

#### Local Composition or Composition Profile of Patterns

It has been observed in Figures 3, 4, 5, and 6 that certain types of residues are more abundant in MIRs patterns (e.g., Ser, Thr, Asn, Asp, Tyr). Thus it's possible to discriminate MIRs and non-MIRs patterns based on their composition. Based on this observation we used a new strategy for converting patterns in numbers. In this case we compute composition of each pattern and represent a pattern by a vector of dimension 21. This is called local composition or composition profile of pattern (CPP), see Ansari and Raghava [31] for detail. We developed CPP based SVM models and achieved a maximum MCC of 0.61 for a pattern of length 17-residues. It was interesting to note that the performance of composition based SVM model is significantly higher than SVM models developed using binary or PSSM profile. We also developed CPP based SVM models using different windows lengths (Table 2). These results clearly indicate that this newly introduced CPP based SVM models are more accurate in prediction of mannose interacting residues (Table S2 & S3).

**Table 2:** The performance of composition based SVM model developed on main dataset using window length 21, 23 and 25 (complete tables shown in Tables S2 & S3).

21 Window					23 Window				25 Window			
Thes	Sen	Spe	Acc	MCC	Sen	Spe	Acc	MCC	Sen	Spe	Acc	MCC
−0.3	91.55	50.53	71.04	0.46	96.60	41.59	69.10	0.46	96.31	37.03	66.67	0.41
−0.2	86.69	70.46	78.57	0.58	93.78	55.00	74.39	0.53	93.68	48.59	71.14	0.47
−0.1	83.87	82.02	82.94	0.66	89.99	72.89	81.44	0.64	90.48	66.67	78.57	0.59
**0**	**83.87**	**82.02**	**82.94**	**0.66**	86.78	82.80	84.79	0.70	87.17	77.07	82.12	0.65
0.1	75.90	92.52	84.21	0.69	**83.09**	**89.21**	**86.15**	**0.72**	84.94	83.87	84.40	0.69
0.2	71.53	94.66	83.09	0.68	80.37	92.91	86.64	0.74	**81.92**	**88.63**	**85.28**	**0.71**
0.3	65.99	95.92	80.95	0.65	76.48	94.85	85.67	0.73	77.45	91.93	84.69	0.70

Bold values indicate the point where sensitivity and specificity is equal or minimum difference with maximum MCC.

#### CPP based SVM Models on Realistic Dataset

**Figure 7:**
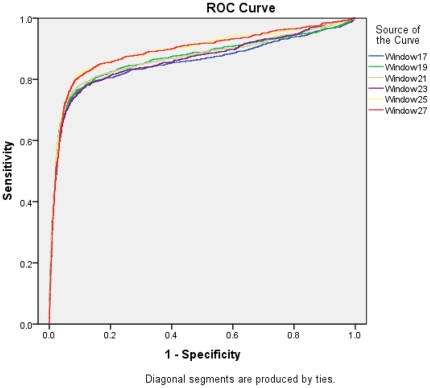
ROC plot for composition based SVM modules at different windows length.

All above model developed on main dataset where MIRs and non-MIRs are equal. In real life there is only few mannose interacting residues in protein thus we developed composition based SVM model on realistic dataset where non-MIRs patterns are 10 times of MIR patterns. On this dataset we achieved maximum MCC 0.58 at window length 25 where sensitivity and specificity are nearly same (Table 3). We achieved maximum MCC 0.62 with 93.72% accuracy at threshold −0.2 MCC was maximum but sensitivity was very poor (Table S4). At threshold −0.7 we achieved balanced performance with sensitivity and specificity (MCC was 0.54 with 89.02% accuracy). In order to understand the performance of models on realistic dataset we have also evaluated our composition based SVM model using threshold independent parameter AUC. As shown in Table 4, we achieved maximum AUC 0.894 at window length 25 (Figure 7).

**Table 3:** The performance of composition based SVM model developed on realistic dataset using different window lengths (complete data in Table S4).

Window Lengths	Thes	Sen	Spe	Acc	MCC
**17**	−0.7	75.61	91.07	89.66	0.54
**19**	−0.7	76.68	90.48	89.22	0.53
**21**	−0.7	77.75	90.52	89.36	0.54
**23**	−0.7	80.27	89.89	89.02	0.54
**25**	−0.7	69.39	94.37	92.10	0.58

**Table 4:** The performance of composition based SVM models in term of AUC on realistic dataset.

Window Lengths	SVM parameter	AUC
**17**	g:0.01 c:2 j:2*	0.855
**19**	g:0.01 c:1 j:2	0.868
**21**	g:0.01 c:1 j:2	0.869
**23**	g:0.01 c:1 j:2	0.863
**25**	g:0.01 c:1 j:1	0.894

*SVM parameters, RBF kernal (g), trade-off between training error & margin (c), cost-factor (j).

### Comparison with Existing Methods

This is important to compare newly developed method with existing methods in order to understand its novelty. Recently, Nasif et al. [22] compare performance of carbohydrate binding methods mainly developed for predicting glucose and galactose binding sites (See Table XI of Nasif *et al.* [22]). Best of authors knowledge, no method has been developed in past for predicting mannose interacting residues in protein from their primary sequence. Thus it is difficult to compare our method directly with any existing method.

### Description of Web Server

The prediction method described in this paper is implemented in the form of a web-server PreMieR (http://www.imtech.res.in/raghava/premier). This server is launch from a Solaris based SUN server using Apache. The common gateway interface (CGI) scripts of server were written in PERL. This server allows users to predict MIRs using compositional profile based SVM models with different threshold range from −1 – +1. The prediction results are presented in graphical form where the predicted MIRs and non-MIRs are displayed in different color.

## Discussion

Mannose binding proteins play an important role in the innate immune response by binding to carbohydrates on the surface of a wide range of pathogens and activate the complement system [24]. Experimental techniques of identification of mannose interacting residue are costly and time consuming. There is a need to develop *in silico* techniques for predicting protein-mannose interaction in order to understand function of MBPs and their role in innate immunity [2]–[4]. In past, methods have been developed for predicting glucose, galactose and carbohydrate interacting residues in a protein [1], [16]–[22] but no method has been developed for predicting mannose interacting residues. In this direction, we had made a systematic attempt to develop an accurate and robust method for predicting MIRs in protein sequences.

In this study, we created clean and standard dataset from SuperSite documentation and PDB and assign MIRs using program LPC [29], [30]. This dataset have 125 non-redundant MBPs where no two MBPs have more than 40% similarity. In order to understand preference of residues in mannose interaction we compute and compare composition of MIRs and non-MIRs (Figures 3, 4, 5, 6). It was observed that certain types of residues are more preferred in mannose interaction than others. It was observed MIRs neighbor residues are also different then non-MIRs neighbor residues. It indicates that mannose interacting sites/pockets are highly conserved. This was also observed that mannose-protein interaction is different than DNA or RNA protein interaction in term of residues preferred interaction [35].

SVM model based on binary patterns of amino acid sequence has been developed to predict mannose interacting residues with low accuracy around 59%. It has been shown in previous studies that evolutionary information of a protein contains more information than single amino acid sequence of protein. In order to improve performance of our models, we used evolutionary information in form of PSSM profile for developing SVM models for predicting mannose interacting residues (Table 1). The accuracy of SVM modules increase significantly from 59% to 66%, it is expected PSSM provides more information than single sequence. During analysis of MIRs, it was observed that residues involved in mannose interaction as well as MIRs neighbors' residues are dominated by certain types of residues. Based on this observation, we used composition profile of patterns (CPP) for developing modules for predicting MIRs instead of binary or PSSM profile. As shown in Table 1 and 2, CPP based SVM modules predict MIRs with high accuracy around 85%. The performance of SVM modules based on CPP is significantly higher than SVM modules based on BPP or PPP. Previously, our group used this concept for predicting conformational B-cell epitopes in proteins.

This is interesting that models based on simple composition of patterns perform better than models based on binary or PSSM profile of patterns. BPP provides more comprehensive information than CPP. In case of BPP, information includes order and types of residues in a pattern, where as CPP contain only composition of residues. Ideally BPP based modules should be more accurate than CPP based modules as it have more information. In real life results are contradictory. This problem may be compared with problem of sub-cellular localization of methods where simple composition based SVM modules out perform alignment based methods like BLAST [36], [37]. Biologically, it is difficult to justify that composition based method can perform better than BPP or PPP based methods. We feel it is due to limitations of representation of patterns to be used in SVM. In case of BPP, pattern of residues N are represented with matrix of N×21 which contain value 1.0 for N elements and 0.0 for N×20. In simple term, values of most of matrix elements are zero, thus it is difficult for any machine learning technique to learn from matrix having most of elements zero. In case of CPP, pattern is presented by only 21 values where most of values are non-zero. This is probable reason that composition based methods is becoming popular over the years [38]. This study will be useful for researcher working in the filed of immunology to understand host pathogen interaction and response of innate immunity.

## Supporting Information

Table S1 **The performance of SVM model using Binary, evolutionary, Compositional information on main dataset.** All supplementary tables are available at http://www.imtech.res.in/raghava/premier/data.php.
(DOC)

Table S2 **The performance of SVM model on 21, 23 and 25 window size using compositional profile on realistic dataset.**
(DOC)

Table S3 **The performance of SVM model using Compositional profile on 19, 21, and 23 window size on main Dataset.**
(DOC)

Table S4 **The performance of SVM model using Compositional profile on 25 window size on main Dataset.**
(DOC)

Datasheet S1 **Performance of Blast.**
(DOC)

## References

[1] Taroni C, Jones S, Thornton JM (2000). Analysis and prediction of carbohydrate binding sites.. Protein Eng.

[2] Sompayrac L (1999). How the Immune System Works.. Blackwell Science.

[3] Koch A, Melbye M, Sorensen P, Homoe P (2001). Acute respiratory tract infections and mannose-binding lectin insufficiency during early childhood.. JAMA.

[4] Larsen F, Madsen HO, Sim RB, Koch C, Garred P (2004). Disease-associated mutations in human mannose-binding lectin compromise oligomerization and activity of the final protein.. J Biol Chem.

[5] Hakomori S (1991). Possible functions of tumor-associated carbohydrate antigens.. Current Opinion in Immunology.

[6] Rashid M, Ramasamy S, Raghava GP (2010). A simple approach for predicting protein-protein interactions.. Curr Protein Pept Sci.

[7] Mishra NK, Raghava GP (2010). Prediction of FAD interacting residues in a protein from its primary sequence using evolutionary information.. BMC Bioinformatics.

[8] Chauhan JS, Mishra NK, Raghava GP (2009). Identification of ATP binding residues of a protein from its primary sequence.. BMC Bioinformatics.

[9] Kumar M, Gromiha MM, Raghava GP (2008). Prediction of RNA binding sites in a protein using SVM and PSSM profile.. Proteins.

[10] Jeong E, Miyano SA (2006). Weighted profile based method for protein-RNA interacting residue prediction.. Lecture notes in computer science.

[11] Bhardwaj N, Lu H (2007). Residue-level prediction of DNA-binding sites and its application on DNA-binding proteins.. FEBS Lett.

[12] Kuznetsov IB, Gou Z, Li R, Hwang S (2006). Using evolutionary and structural information to predict DNA-binding sites on DNA-binding proteins.. Proteins.

[13] Ahmad S, Gromiha MM, Sarai A (2004). Analysis and prediction of DNA-binding proteins and their binding residues based on composition, sequence and structural information.. Bioinformatics.

[14] Julenius K, Mølgaard A, Gupta R, Brunak S (2005). Prediction, conservation analysis, and structural characterization of mammalian mucin-type O-glycosylation sites.. Glycobiology.

[15] Rao VSR, Lam K, Qasba PK (1998). Architecture of the sugar binding sites in carbohydrate binding proteins—a computer modeling study.. Int J Biol Macromol.

[16] Shionyu-Mitsuyama C, Shirai T, Ishida H, Yamane T (2003). An empirical approach for structure-based prediction of carbohydrate-binding sites on proteins.. Protein Eng.

[17] Kulharia M, Bridgett SJ, Goody RS, Jackson RM (2009). InCa-SiteFinder: a method for structure-based prediction of inositol and carbohydrate binding sites on proteins.. J Mol Graph Model.

[18] Patra M, Mandal C (2006). Search for glucose/galactose-binding proteins in newly discovered protein sequences using molecular modeling techniques and structural analysis.. Glycobiology.

[19] Sujatha MS, Balaji PV (2004). Identification of common structural features of binding sites in galactose-specific proteins.. Protein Struct Funct Bioinf.

[20] Malik A, Ahmad S (2007). Sequence and structural features of carbohydrate binding in proteins and assessment of predictability using a neural network.. BMC Structural Biology.

[21] Malik A, Firoz A, Jha V, Ahmad S (2010). PROCARB: A Database of Known and Modelled Carbohydrate-Binding Protein Structures with Sequence-Based Prediction Tools.. Adv Bioinformatics.

[22] Nassif H, Al-Ali H, Khuri S, Keirouz W (2009). Prediction of protein-glucose binding sites using support vector machines.. Proteins.

[23] Bouwman LH, Roep BO, Roos A (2006). Mannose-binding lectin: clinical implications for infection, transplantation, and autoimmunity.. Hum Immunol.

[24] Larsen F, Madsen HO, Sim RB, Koch C, Garred P (2004). Disease-associated mutations in human mannose-binding lectin compromise oligomerization and activity of the final protein.. J Biol Chem.

[25] Altschul SF, Madden TL, Schaffer AA, Zhang J, Zhang Z (1997). Gapped BLAST and PSI-BLAST: a new generation of protein database search programs.. Nucleic Acids Res.

[26] Joachims T (1999). Making large scale SVM learning practical.. Advances in kernel methods: Support Vector Learning:.

[27] Kaur H, Raghava GP (2003). Prediction of beta-turns in proteins from multiple alignment using neural network.. Protein Sci.

[28] Kaur H, Raghava GP (2004). A neural network method for prediction of beta-turn types in proteins using evolutionary information.. Bioinformatics.

[29] Bauer RA, Gunther S, Jansen D, Heeger C, Thaben PF (2009). SuperSite: dictionary of metabolite and drug binding sites in proteins.. Nucleic Acids Res.

[30] Sobolev V, Sorokine A, Prilusky J, Abola EE, Edelman M (1999). Automated analysis of interatomic contacts in proteins.. Bioinformatics.

[31] Ansari HR, Raghava GP (2010). Identification of conformational B-cell Epitopes in an antigen from its primary sequence.. Immunome Research.

[32] Raghava GP, Han JH (2005). Correlation and prediction of gene expression level from amino acid and dipeptide composition of its protein.. BMC Bioinformatics.

[33] Quiocho FA (1989). Protein-carbohydrate interactions: basic molecular features.. Pure & Appl Chem.

[34] Muraki M (2002). The importance of CH/pi interactions to the function of carbohydrate binding proteins.. Protein Pept Lett.

[35] Gromiha MM (1999). A simple method for predicting transmembrane alpha helices with better accuracy.. Protein Eng.

[36] Rashid M, Saha S, Raghava GP (2007). Support Vector Machine-based method for predicting subcellular localization of mycobacterial proteins using evolutionary information and motifs.. BMC Bioinformatics.

[37] Garg A, Raghava GP (2008). ESLpred2 improved method for predicting subcellular localization of eukaryotic proteins.. BMC Bioinformatics.

[38] Kumar M, Thakur V, Raghava GP (2008). COPid: composition based protein identification.. In Silico Biol.

