# Supplementary material for: Identification of Mannose Interacting Residues Using Local Composition
Source: PLoS One. 2011 Sep 13;6(9):e24039. doi: 10.1371/journal.pone.0024039 (PMC3172211; doi:10.1371/journal.pone.0024039)
Supplement: Table S1 — The performance of SVM model using Binary, evolutionary, Compositional information on main dataset. All supplementary tables are available at http://www.imtech.res.in/raghava/premier/data.php. (DOC) [file pone.0024039.s001.doc]

# Supplementary data

**Table S1:** The performance of SVM model developed on main dataset using Binary, Evolutionary and Compositional profile.

| **17 Window** | | | | | | | | | | | | |
| --- | --- | --- | --- | --- | --- | --- | --- | --- | --- | --- | --- | --- |
| **Binary** | | | | | **PSSM** | | | | **Composition** | | | |
| **Thes** | **Sen** | **Spe** | **Acc** | **MCC** | **Sen** | **Spe** | **Acc** | **MCC** | **Sen** | **Spe** | **Acc** | **MCC** |
| -1.0 | 99.59 | 1.94 | 50.77 | 0.07 | 97.89 | 9.47 | 53.68 | 0.16 | 100 | 0.29 | 50.15 | 0.04 |
| -0.9 | 99.08 | 3.58 | 51.33 | 0.09 | 97.28 | 11.78 | 54.53 | 0.17 | 99.9 | 1.96 | 50.93 | 0.09 |
| -0.8 | 98.37 | 5.41 | 51.89 | 0.1 | 96.48 | 14.4 | 55.44 | 0.19 | 99.9 | 1.96 | 50.93 | 0.09 |
| -0.7 | 96.94 | 7.76 | 52.35 | 0.1 | 94.96 | 17.62 | 56.29 | 0.2 | 99.71 | 2.44 | 51.08 | 0.09 |
| -0.6 | 93.87 | 11.03 | 52.45 | 0.09 | 93.55 | 21.85 | 57.7 | 0.22 | 99.71 | 8.11 | 53.91 | 0.09 |
| -0.5 | 91.42 | 16.14 | 53.78 | 0.11 | 90.84 | 26.69 | 58.76 | 0.23 | 98.83 | 29.72 | 64.27 | 0.19 |
| -0.4 | 87.33 | 23.08 | 55.21 | 0.14 | 88.12 | 31.42 | 59.77 | 0.24 | 98.14 | 37.34 | 67.74 | 0.39 |
| -0.3 | 81.21 | 30.34 | 55.77 | 0.13 | 85.6 | 36.76 | 61.18 | 0.26 | 96.77 | 41.64 | 69.21 | 0.46 |
| -0.2 | 73.44 | 39.43 | 56.44 | 0.14 | 82.58 | 43.91 | 63.24 | 0.29 | 93.84 | 48.39 | 71.11 | 0.47 |
| -0.1 | 66.09 | 49.44 | 57.76 | 0.16 | 78.05 | 50.15 | 64.1 | 0.29 | 87 | 66.47 | 76.74 | 0.55 |
| **0** | **58.43** | **60.78** | **59.6** | **0.19** | 73.51 | 57.8 | 65.66 | 0.32 | **77.03** | **82.89** | **79.96** | **0.60** |
| 0.1 | 49.74 | 68.54 | 59.14 | 0.19 | **67.37** | **63.54** | **65.46** | **0.31** | 68.82 | 90.13 | 79.47 | 0.60 |
| 0.2 | 40.65 | 75.89 | 58.27 | 0.18 | 59.01 | 68.68 | 63.85 | 0.28 | 63.44 | 94.62 | 79.03 | 0.61 |
| 0.3 | 31.56 | 82.89 | 57.2 | 0.17 | 51.56 | 73.82 | 62.69 | 0.26 | 56.79 | 96.48 | 76.64 | 0.58 |
| 0.4 | 23.8 | 87.84 | 55.82 | 0.15 | 44.11 | 78.95 | 61.53 | 0.25 | 49.36 | 97.26 | 73.31 | 0.53 |
| 0.5 | 18.28 | 90.91 | 54.6 | 0.13 | 36.76 | 84.49 | 60.62 | 0.24 | 35.29 | 98.83 | 67.06 | 0.44 |
| 0.6 | 12.16 | 94.38 | 53.27 | 0.11 | 30.31 | 87.81 | 59.06 | 0.22 | 24.44 | 99.22 | 61.83 | 0.36 |
| 0.7 | 8.99 | 96.42 | 52.71 | 0.11 | 23.26 | 91.54 | 57.4 | 0.2 | 16.62 | 99.51 | 58.06 | 0.29 |
| 0.8 | 5.62 | 97.55 | 51.58 | 0.08 | 16.62 | 94.56 | 55.59 | 0.18 | 9.97 | 99.71 | 54.84 | 0.22 |
| 0.9 | 3.47 | 98.67 | 51.07 | 0.07 | 13.6 | 95.77 | 54.68 | 0.16 | 7.14 | 99.9 | 53.52 | 0.19 |
| 1.0 | 1.94 | 99.08 | 50.51 | 0.04 | 9.37 | 98.09 | 53.73 | 0.16 | 3.81 | 99.9 | 51.86 | 0.13 |

* Bold values indicate the point where sensitivity and specificity is equal or minimum difference with highest MCC.
