# Supplementary material for: Identification of Mannose Interacting Residues Using Local Composition
Source: PLoS One. 2011 Sep 13;6(9):e24039. doi: 10.1371/journal.pone.0024039 (PMC3172211; doi:10.1371/journal.pone.0024039)
Supplement: Table S2 — The performance of SVM model on 21, 23 and 25 window size using compositional profile on realistic dataset. (DOC) [file pone.0024039.s002.doc]

# Supplementary data

**Table S2:** The performance of composition based SVM model on main datset using window length 17, 19 and 21.

| **17 Window** | | | | | **19 Window** | | | | **21 Window** | | | |
| --- | --- | --- | --- | --- | --- | --- | --- | --- | --- | --- | --- | --- |
| **Thes** | **Sen** | **Spe** | **Acc** | **MCC** | **Sen** | **Spe** | **Acc** | **MCC** | **Sen** | **Spe** | **Acc** | **MCC** |
| -1.0 | 100 | 0.29 | 50.15 | 0.04 | 100.00 | 0.29 | 50.15 | 0.04 | 100.00 | 0.68 | 50.34 | 0.06 |
| -0.9 | 99.9 | 1.96 | 50.93 | 0.09 | 100.00 | 0.87 | 50.44 | 0.07 | 99.71 | 1.17 | 50.44 | 0.05 |
| -0.8 | 99.9 | 1.96 | 50.93 | 0.09 | 99.90 | 1.46 | 50.68 | 0.08 | 99.51 | 2.72 | 51.12 | 0.09 |
| -0.7 | 99.71 | 2.44 | 51.08 | 0.09 | 99.51 | 4.18 | 51.85 | 0.12 | 99.22 | 10.98 | 55.10 | 0.22 |
| -0.6 | 99.71 | 8.11 | 53.91 | 0.09 | 99.22 | 11.47 | 55.34 | 0.22 | 98.83 | 17.69 | 58.26 | 0.28 |
| -0.5 | 98.83 | 29.72 | 64.27 | 0.19 | 98.64 | 16.62 | 57.63 | 0.27 | 98.15 | 21.67 | 59.91 | 0.31 |
| -0.4 | 98.14 | 37.34 | 67.74 | 0.39 | 97.96 | 21.28 | 59.62 | 0.30 | 97.47 | 26.14 | 61.81 | 0.34 |
| -0.3 | 96.77 | 41.64 | 69.21 | 0.46 | 95.92 | 28.96 | 62.44 | 0.33 | 95.63 | 33.14 | 64.38 | 0.37 |
| -0.2 | 93.84 | 48.39 | 71.11 | 0.47 | 93.39 | 44.80 | 69.10 | 0.44 | 91.55 | 50.53 | 71.04 | 0.46 |
| -0.1 | 87 | 66.47 | 76.74 | 0.55 | 87.66 | 71.62 | 79.64 | 0.60 | 86.69 | 70.46 | 78.57 | 0.58 |
| **0** | **77.03** | **82.89** | **79.96** | **0.60** | **82.80** | **84.65** | **83.72** | **0.67** | **83.87** | **82.02** | **82.94** | **0.66** |
| 0.1 | 68.82 | 90.13 | 79.47 | 0.60 | 77.36 | 91.74 | 84.55 | 0.70 | 80.08 | 88.82 | 84.45 | 0.69 |
| 0.2 | 63.44 | 94.62 | 79.03 | 0.61 | 71.33 | 93.59 | 82.46 | 0.67 | 75.90 | 92.52 | 84.21 | 0.69 |
| 0.3 | 56.79 | 96.48 | 76.64 | 0.58 | 65.99 | 95.43 | 80.71 | 0.64 | 71.53 | 94.66 | 83.09 | 0.68 |
| 0.4 | 49.36 | 97.26 | 73.31 | 0.53 | 60.54 | 96.60 | 78.57 | 0.61 | 65.99 | 95.92 | 80.95 | 0.65 |
| 0.5 | 35.29 | 98.83 | 67.06 | 0.44 | 49.66 | 97.47 | 73.57 | 0.54 | 58.31 | 96.99 | 77.65 | 0.60 |
| 0.6 | 24.44 | 99.22 | 61.83 | 0.36 | 38.58 | 98.45 | 68.51 | 0.46 | 45.09 | 97.76 | 71.43 | 0.50 |
| 0.7 | 16.62 | 99.51 | 58.06 | 0.29 | 29.06 | 99.22 | 64.14 | 0.40 | 34.89 | 98.35 | 66.62 | 0.43 |
| 0.8 | 9.97 | 99.71 | 54.84 | 0.22 | 19.73 | 99.42 | 59.57 | 0.32 | 26.14 | 99.13 | 62.63 | 0.37 |
| 0.9 | 7.14 | 99.9 | 53.52 | 0.19 | 12.83 | 99.81 | 56.32 | 0.26 | 14.77 | 99.32 | 57.05 | 0.26 |
| 1.0 | 3.81 | 99.9 | 51.86 | 0.13 | 5.64 | 100.00 | 52.82 | 0.17 | 6.51 | 99.61 | 53.06 | 0.17 |

* Bold values indicate the point where sensitivity and specificity is equal or minimum difference with highest MCC.
