# Supplementary material for: Identification of Mannose Interacting Residues Using Local Composition
Source: PLoS One. 2011 Sep 13;6(9):e24039. doi: 10.1371/journal.pone.0024039 (PMC3172211; doi:10.1371/journal.pone.0024039)
Supplement: Table S3 — The performance of SVM model using Compositional profile on 19, 21, and 23 window size on main Dataset. (DOC) [file pone.0024039.s003.doc]

# Supplementary data

**Table S3:** The performance of composition based SVM model using window length 23 and 25 on main Dataset.

| **23 Window** | | | | | **25 Window** | | | |
| --- | --- | --- | --- | --- | --- | --- | --- | --- |
| **Thes** | **Sen** | **Spe** | **Acc** | **MCC** | **Sen** | **Spec** | **Acc** | **MCC** |
| -1.0 | 100.00 | 0.68 | 50.34 | 0.06 | 100.00 | 0.68 | 50.34 | 0.06 |
| -0.9 | 99.90 | 2.33 | 51.12 | 0.10 | 99.81 | 2.82 | 51.31 | 0.11 |
| -0.8 | 99.71 | 5.73 | 52.72 | 0.16 | 99.71 | 7.29 | 53.50 | 0.18 |
| -0.7 | 99.42 | 19.34 | 59.38 | 0.31 | 99.61 | 16.33 | 57.97 | 0.29 |
| -0.6 | 98.93 | 25.17 | 62.05 | 0.36 | 98.93 | 21.38 | 60.16 | 0.32 |
| -0.5 | 98.35 | 29.35 | 63.85 | 0.38 | 98.74 | 25.66 | 62.20 | 0.36 |
| -0.4 | 97.38 | 33.43 | 65.40 | 0.40 | 97.76 | 30.03 | 63.90 | 0.38 |
| -0.3 | 96.60 | 41.59 | 69.10 | 0.46 | 96.31 | 37.03 | 66.67 | 0.41 |
| -0.2 | 93.78 | 55.00 | 74.39 | 0.53 | 93.68 | 48.59 | 71.14 | 0.47 |
| -0.1 | 89.99 | 72.89 | 81.44 | 0.64 | 90.48 | 66.67 | 78.57 | 0.59 |
| 0 | 86.78 | 82.80 | 84.79 | 0.70 | 87.17 | 77.07 | 82.12 | 0.65 |
| 0.1 | **83.09** | **89.21** | **86.15** | **0.72** | 84.94 | 83.87 | 84.40 | 0.69 |
| 0.2 | 80.37 | 92.91 | 86.64 | 0.74 | **81.92** | **88.63** | **85.28** | **0.71** |
| 0.3 | 76.48 | 94.85 | 85.67 | 0.73 | 77.45 | 91.93 | 84.69 | 0.70 |
| 0.4 | 69.68 | 96.21 | 82.94 | 0.68 | 73.76 | 94.56 | 84.16 | 0.70 |
| 0.5 | 62.68 | 97.08 | 79.88 | 0.64 | 68.42 | 96.11 | 82.26 | 0.67 |
| 0.6 | 53.16 | 97.57 | 75.36 | 0.57 | 60.84 | 96.79 | 78.81 | 0.62 |
| 0.7 | 41.30 | 98.35 | 69.83 | 0.48 | 49.08 | 97.96 | 73.52 | 0.54 |
| 0.8 | 31.10 | 99.13 | 65.11 | 0.41 | 37.80 | 98.64 | 68.22 | 0.46 |
| 0.9 | 20.41 | 99.51 | 59.96 | 0.33 | 25.27 | 99.03 | 62.15 | 0.36 |
| 1.0 | 9.43 | 99.81 | 54.62 | 0.22 | 10.50 | 99.61 | 55.05 | 0.22 |

* Bold values indicate the point where sensitivity and specificity is equal or minimum difference with highest MCC.
