# Supplementary material for: Identification of Mannose Interacting Residues Using Local Composition
Source: PLoS One. 2011 Sep 13;6(9):e24039. doi: 10.1371/journal.pone.0024039 (PMC3172211; doi:10.1371/journal.pone.0024039)
Supplement: Table S4 — The performance of SVM model using Compositional profile on 25 window size on main Dataset. (DOC) [file pone.0024039.s004.doc]

# Supplementary data

**Table S4:** The performance of composition based SVM models on Realistic Dataset using window length 21, 23 and 25.

| **21 Window** | | | | | **23 Window** | | | | **25 Window** | | | |
| --- | --- | --- | --- | --- | --- | --- | --- | --- | --- | --- | --- | --- |
| **Thes** | **Sen** | **Spe** | **Acc** | **MCC** | **Sen** | **Spe** | **Acc** | **MCC** | **Sen** | **Spe** | **Acc** | **MCC** |
| -1.0 | 92.52 | 28.77 | 34.52 | 0.14 | 93.59 | 35.54 | 40.81 | 0.18 | 91.25 | 44.59 | 48.83 | 0.21 |
| -0.9 | 85.42 | 67.13 | 68.78 | 0.31 | 86.01 | 70.26 | 71.69 | 0.34 | 81.54 | 83.00 | 82.87 | 0.44 |
| -0.8 | 81.05 | 84.40 | 84.10 | 0.46 | 82.80 | 84.66 | 84.49 | 0.47 | 75.51 | 91.87 | 90.39 | 0.55 |
| -0.7 | **77.75** | **90.52** | **89.36** | **0.54** | **80.27** | **89.89** | **89.02** | **0.54** | **69.39** | **94.37** | **92.10** | **0.58** |
| -0.6 | 74.64 | 93.13 | 91.46 | 0.58 | 77.07 | 92.56 | 91.15 | 0.58 | 63.65 | 95.86 | 92.94 | 0.58 |
| -0.5 | 72.11 | 94.38 | 92.37 | 0.59 | 74.25 | 94.15 | 92.35 | 0.60 | 58.41 | 96.84 | 93.35 | 0.58 |
| -0.4 | 68.80 | 95.45 | 93.04 | 0.60 | 71.33 | 95.25 | 93.08 | 0.62 | 51.60 | 97.43 | 93.27 | 0.55 |
| -0.3 | 66.47 | 96.10 | 93.43 | 0.61 | 67.35 | 96.10 | 93.49 | 0.62 | 45.87 | 97.85 | 93.13 | 0.52 |
| -0.2 | 62.39 | 96.72 | 93.62 | 0.60 | 64.43 | 96.65 | 93.72 | 0.62 | 41.11 | 98.25 | 93.06 | 0.50 |
| -0.1 | 58.21 | 97.28 | 93.76 | 0.60 | 60.93 | 97.02 | 93.74 | 0.61 | 37.12 | 98.64 | 93.05 | 0.49 |
| 0 | 55.10 | 97.70 | 93.86 | 0.59 | 55.69 | 97.39 | 93.60 | 0.58 | 33.62 | 98.98 | 93.05 | 0.48 |
| 0.1 | 50.15 | 97.97 | 93.66 | 0.56 | 51.80 | 97.77 | 93.59 | 0.57 | 29.64 | 99.15 | 92.83 | 0.45 |
| 0.2 | 44.02 | 98.37 | 93.47 | 0.53 | 46.74 | 98.01 | 93.35 | 0.54 | 25.46 | 99.28 | 92.58 | 0.42 |
| 0.3 | 38.39 | 98.78 | 93.33 | 0.51 | 41.30 | 98.35 | 93.17 | 0.51 | 22.06 | 99.50 | 92.47 | 0.40 |
| 0.4 | 32.07 | 99.09 | 93.05 | 0.47 | 36.54 | 98.66 | 93.02 | 0.49 | 18.66 | 99.59 | 92.24 | 0.37 |
| 0.5 | 27.41 | 99.28 | 92.80 | 0.44 | 31.20 | 98.96 | 92.81 | 0.45 | 16.42 | 99.68 | 92.12 | 0.35 |
| 0.6 | 23.62 | 99.44 | 92.61 | 0.41 | 27.11 | 99.16 | 92.61 | 0.43 | 12.93 | 99.76 | 91.87 | 0.31 |
| 0.7 | 18.66 | 99.60 | 92.30 | 0.37 | 21.96 | 99.36 | 92.33 | 0.39 | 10.40 | 99.84 | 91.72 | 0.28 |
| 0.8 | 12.44 | 99.70 | 91.84 | 0.30 | 17.10 | 99.56 | 92.07 | 0.35 | 7.09 | 99.89 | 91.47 | 0.23 |
| 0.9 | 8.84 | 99.83 | 91.63 | 0.26 | 11.18 | 99.74 | 91.69 | 0.28 | 4.86 | 99.93 | 91.30 | 0.19 |
| 1.0 | 4.86 | 99.93 | 91.36 | 0.19 | 5.64 | 99.86 | 91.30 | 0.20 | 2.62 | 99.99 | 91.15 | 0.15 |

* Bold values indicate the point where sensitivity and specificity is equal or minimum difference with highest MCC.
