# Supplementary material for: Identification of Mannose Interacting Residues Using Local Composition
Source: PLoS One. 2011 Sep 13;6(9):e24039. doi: 10.1371/journal.pone.0024039 (PMC3172211; doi:10.1371/journal.pone.0024039)
Supplement: Datasheet S1 — Performance of Blast. (DOC) [file pone.0024039.s005.doc]

# Supplementary data

**Performance of BLAST**

In order to evaluate performance of BLAST, we used 120 mannose binding proteins. NCBI-BLAST was run locally for each query protein sequence against remaining protein in dataset and out of 120 proteins we got 40 blast hit only for 40 proteins, among those we are giving the detailed description of 12 Mannose binding proteins which have maximum E-value and has more than three mannose interacting residues. Alignment details (BLAST) of each protein are shown here. Each mannose interacting residues is mapped on the alignment. Overlapped True positive residues are highlighted, counted and equation for the calculation of Sensitivity and PPV is also mentioned.

| **Query** | **Target** | **E-value** | **Mannose**  **Interacting residues in query** | **Mannose interacting residues in target** | **Blast Local Alignment** | | |
| --- | --- | --- | --- | --- | --- | --- | --- |
|  |  |  |  |  | **TP** | **Sen %** | **PPV %** |
| 1B5F_A | 1DP5_A | 2e-56 | 05 | 12 | 02 | 40.0 | 16.67 |
| 1CVN_A | 1QMO_E | 9e-41 | 13 | 06 | 05 | 38.46 | 83.33 |
| 1CXP_C | 2E9E_A | e-160 | 08 | 04 | 00 | 00.0 | 00.0 |
| 1IA5_A | 1RMG_A | 5e-05 | 32 | 57 | 00 | 00.0 | 00.0 |
| 1Q8O_A | 1BQP_A | e-149 | 17 | 09 | 09 | 52.94 | 100 |
| 2E22_A | 1HM2_A | 3e-37 | 13 | 09 | 00 | 00.0 | 00.0 |
| 2OK5_A | 1GPZ_A | 8e-12 | 04 | 01 | 00 | 00.0 | 00.0 |
| 1GPE_A | 1JU2_A | 1e-15 | 07 | 04 | 00 | 00.0 | 00.0 |
| 1KDG_A | 1GPE_A | 1e-09 | 04 | 07 | 00 | 00.0 | 00.0 |
| 1O0V_A | 1HZH_H | 2e-34 | 11 | 03 | 01 | 9.09 | 33.3 |
| 1QMO_A | 1Q8O_A | 5e-23 | 05 | 17 | 02 | 40 | 11.76 |
| 1R46_A | 1SZN_A | 3e-47 | 04 | 12 | 01 | 25 | 8.33 |
| **Average** |  |  |  |  |  | **17.12** | **21.11** |

**TP** = Mannose interacting residues common in both

**Sen % =** % Sensitivity

**PPV %** = % Probability of correct positive prediction

**Definitions:**

- MIRs = Mannose interacting Residues
- True Positive= number of common MIRs (highlighted yellow); MIRs which were actually positive and predicted as positive in target alignment
- False Negative= MIRs which were actually positive but predicted as negative in target alignment
- True Negative= MIRs which were actually negative and predicted as negative in target alignment
- False Positive= MIRs which were actually negative but predicted as positive in target alignment
- (true positive + false negative)= actual MIRs in query
- (true positive + false positive)= total positive prediction in query = total MIRs in target
- **Calculation of Sensitivity:**

Sensitivity = True Positive / (true positive + false negative)

Or sensitivity = number of common residues/MIRs in query

**% sensitivity** = (12/26)*100 = **46.2**

- **Calculation of Probability of correct positive prediction (PPV):**

PPV = True Positive/ (true positive + false positive)

= True Positive/ total positive prediction

= number of common residues/MIRs in target

**% PPV** = (12/24)*100 = **50**

Query= 1B5F:A(239 letters)

>1B5F:A

GSAVVALTNDRDTSYFGEIGIGTPPQKFTVIFDTGSSVLWVPSSKCINSKACRAHSMYESSDSSTYKENGTFGAIIYGTGSITGFFSQDSVTIGDLVVKEQDFIEATDEADNVFLHRLFDGILGLS**f**QTISVPV**w**YNM**ln**QG**l**VKERRFSFWLNRNVDEEEGGELVFGGLDPNHFRGDHTYVPVTYQYYWQFGIGDVLIGDKSTGFCAPGCQAFADSGTSLLSGPTAIVTQINHAIGAN

Top Hit= 1DP5:A; Length = 153

>1DP5:A

GGHDVPLTNYLNAQYYTDITLGTPPQNFKVILDTGSSNLWVPSNECGSLACFLHSKYDHEASSSYKANGTEFAIQYGTGSLEGYISQDTLSIGDLTIPKQDFAEATSEPGLTFAFGKFDGILGLGY**d**TISVD**k**V**v**PPF**yn**A**iq**QDLLDE**k**RFAFYLGDTSKDTENGGEATFGGIDESKFKGDITWLP**v**RRKAYWEVKFEGIGLGDEYAELESHGAAIDTGTSLITLPSGLAEMINAEIGAKKGWTGQYTLDCNTRDNLPDLIFNFNGYNFTIGPYDYTLEVSGSCISAITPMDFPEPVGPLAIVGDAFLRKYYSI**yd**L**g**NNAVGLAKAI

Query: 5 VALTNDRDTSYFGEIGIGTPPQKFTVIFDTGSSVLWVPSSKCINSKACRAHSMYESSDSS 64

V LTN + Y+ +I +GTPPQ F VI DTGSS LWVPS++C S AC HS Y+ SS

Sbjct: 5 VPLTNYLNAQYYTDITLGTPPQNFKVILDTGSSNLWVPSNEC-GSLACFLHSKYDHEASS 63

Query: 65 TYKENGTFGAIIYGTGSITGFFSQDSVTIGDLVVKEQDFIEATDEADNVFLHRLFDGILG 124

+YK NGT AI YGTGS+ G+ SQD+++IGDL + +QDF EAT E F FDGILG

Sbjct: 64 SYKANGTEFAIQYGTGSLEGYISQDTLSIGDLTIPKQDFAEATSEPGLTFAFGKFDGILG 123

Query: 125 LS**F**QTIS----VPV**W**YNM**LN**QG**L**VKERRFSFWL-NRNVDEEEGGELVFGGLDPNHFRGDH 179

L + TIS VP +YN + Q L+ E+RF+F+L + + D E GGE FGG+D + F+GD

Sbjct: 124 LGY**D**TISVD**K**V**V**PPF**YN**A**IQ**QDLLDE**K**RFAFYLGDTSKDTENGGEATFGGIDESKFKGDI 183

Query: 180 TYVPVTYQYYWQFGIGDVLIGDKSTGFCAPGCQAFADSGTSLLSGPTAIVTQINHAIGAN 239

T++PV + YW+ + +GD+ + G A D+GTSL++ P+ + IN IGA

Sbjct: 184 TWLPVRRKAYWEVKFEGIGLGDEYAELESHG--AAIDTGTSLITLPSGLAEMINAEIGAK 241

- **actual MIRs in query** (small red) = 5
- **MIRs in target** (small green)= 12
- **Common MIRs (True positive residues in query which were actually MIRs and also predicted as MIRs in alignment)** (highlighted in yellow) =02

Query= 1CVN_A
>1CVN:A

ADTIVAVELDT**ypntd**IGDPSYPHIGIDIKSVRSKKTAKWNMQNGKVGTAHIIYNSVDKRLSAVVSYPNADSATVSYDVDLDNVLPEWVRVGLSAST**gly**KETNTILSWSFTSKLKSNSTHETNALHFMFNQFSKDQKDLILQGDATTGTDGNLELTRVSSNGSPQGSSVGRALFYAPVHIWESSAVVASFEATFTFLIKSPDSHP**ad**GIAFFISNIDSSIPSGS**tgr**LLGLFPDAN

Top Hit= 1QMO_E

>1QMO:E

SNVVAVEFDT**y**L**n**PD**y**GDPNYIHIGIDVNSIRSKVTAKWDWQNGKIATAHISYNSVSKRLSVTSYYAGSKPATLSYDIELHTVLPEWVRVGLSAST**gqd**KERNTVHSWSFTSSLWTNVAKKENENKYITRGVL

Score = 155 bits (392), Expect = 9e-41, Method: Compositional matrix adjust.

Identities = 77/126 (61%), Positives = 92/126 (73%)

Query: 4 IVAVELDT**YPNTD**IGDPSYPHIGIDIKSVRSKKTAKWNMQNGKVGTAHIIYNSVDKRLSA 63

+VAVE DTY N D GDP+Y HIGID+ S+RSK TAKW+ QNGK+ TAHI YNSV KRLS

Sbjct: 3 VVAVEFDT**Y**L**N**PD**Y**GDPNYIHIGIDVNSIRSKVTAKWDWQNGKIATAHISYNSVSKRLSV 62

Query: 64 VVSYPNADSATVSYDVDLDNVLPEWVRVGLSAST**GLY**KETNTILSWSFTSKLKSNSTHET 123

Y + AT+SYD++L VLPEWVRVGLSASTG KE NT+ SWSFTS L +N +

Sbjct: 63 TSYYAGSKPATLSYDIELHTVLPEWVRVGLSAST**GQD**KERNTVHSWSFTSSLWTNVAKKE 122

Query: 124 NALHFM 129

N ++

Sbjct: 123 NENKYI 128

- **actual MIRs in query** (small red) = 13
- **MIRs in target** (small green)= 06
- **Common MIRs (True positive residues in query which were actually MIRs and also predicted as MIRs in alignment)** (highlighted in yellow) =05

Query= 1CXP_C

1CXP:C::VNCETSCVQQPPCFPLKIPPNDPRIKNQADCIPFFRSCPACPGSNITIRNQINALTSFVDASMVYGSEEPLARNLRNMSNQLGLLAVNQRFQDNGRALLPFDNLHDDPCLLTNRSARIPCFLAGDTRSSEMPELTSMHTLLLREHNRLATELKSLNPRWDGERLYQEARKIVGAMVQIITYRDYLPLVLGPTAMR**ky**LPTYRSYNDSVDPRIANVFTNAFRYGHTLIQPFMFRLDNRYQPMEPNPRVPLSRVFFASWRVVLEGGIDPILRGLMATPAKLNRQNQIAVDEIRERLFEQVMRIGLDLPALNMQRSRDHGLPGYNAWRR**fcg**LPQPETVGQLGTVLRNLKLARKLMEQYGTPNNIDIWMGGVSEPLKRKGRVGPLLA**c**IIG**t**QFR**k**LRDGDRFWWENEGVFSMQQRQALAQISLPRIICDNTGITTVSKNNIFMSNSYPRDFVNCSTLPALNLASWREA

Top Hit= 2E9E_A

2E9E:A::SWEVGCGAPVPLVTCDEQSPYRTITGDCNNRRSPALGAANRALARWLPAEYEDGLAVPFGWTQRKTRNGFRVPLAREVSNKIVGYLDEEGVLDQNRSLLFMQWGQIVDHDLDFAPETELGSSEHSKVQCEEYCVQGDECFPIMFPKNDPKLKTQGKCMPFFRAGFVCPTPPYQSLARDQINAVTSFLDASLVYGSEPSLASRLRNLSSPLGLMAVNQEA**w**DH**g**LAYPPFNNVKPSPCEFINTTAHVPCFQAGDSRASEQILLATVHTLLLREHNRLARELKRLNPHWDGEMLYQEARKILGAFIQIITFRDYLPIVLGSEMQKWIPPYQGYNNSVDPRISNVFTFAFRFGHMEVPSTVSRLDENYQPWG**pe**AELPLHTLFFNTWRIIKDGGIDPLVRGLLAKNSKLMNQNKMVTSELRNKLFQPTHKVHGFDLAAINLQRCRDHGMPGYNSWRGFCGLSQPKTLKGLQAVLKNKVLAKKLLDLYKTPDNIDIWIGGNAEPMVERGRVGPLLACLLGRQFQQIRDGDRFWWENPGVFTEKQRDSLQKVSFSRLICDNTHITKVPLHAFQANNYPHDFVDCSAVDKLDLSPWASREN

Score = 554 bits (1428), Expect = e-160, Method: Compositional matrix adjust.

Identities = 261/467 (55%), Positives = 348/467 (74%), Gaps = 7/467 (1%)

Query: 1 VNCETSCVQQPPCFPLKIPPNDPRIKNQADCIPFFRS---CPACPGSNITIRNQINALTS 57

V CE CVQ CFP+ P NDP++K Q C+PFFR+ CP P ++ R+QINA+TS

Sbjct: 127 VQCEEYCVQGDECFPIMFPKNDPKLKTQGKCMPFFRAGFVCPTPPYQSLA-RDQINAVTS 185

Query: 58 FVDASMVYGSEEPLARNLRNMSNQLGLLAVNQRFQDNGRALLPFDNLHDDPCLLTNRSAR 117

F+DAS+VYGSE LA LRN+S+ LGL+AVNQ D+G A PF+N+ PC N +A

Sbjct: 186 FLDASLVYGSEPSLASRLRNLSSPLGLMAVNQEA**W**DH**G**LAYPPFNNVKPSPCEFINTTAH 245

Query: 118 IPCFLAGDTRSSEMPELTSMHTLLLREHNRLATELKSLNPRWDGERLYQEARKIVGAMVQ 177

+PCF AGD+R+SE L ++HTLLLREHNRLA ELK LNP WDGE LYQEARKI+GA +Q

Sbjct: 246 VPCFQAGDSRASEQILLATVHTLLLREHNRLARELKRLNPHWDGEMLYQEARKILGAFIQ 305

Query: 178 IITYRDYLPLVLGPTAMR**KY**LPTYRSYNDSVDPRIANVFTNAFRYGHTLIQPFMFRLDNR 237

IIT+RDYLP+VLG + M+K++P Y+ YN+SVDPRI+NVFT AFR+GH + + RLD

Sbjct: 306 IITFRDYLPIVLG-SEMQKWIPPYQGYNNSVDPRISNVFTFAFRFGHMEVPSTVSRLDEN 364

Query: 238 YQPMEPNPRVPLSRVFFASWRVVLEGGIDPILRGLMATPAKLNRQNQIAVDEIRERLFEQ 297

YQP P +PL +FF +WR++ +GGIDP++RGL+A +KL QN++ E+R +LF+

Sbjct: 365 YQPWG**PE**AELPLHTLFFNTWRIIKDGGIDPLVRGLLAKNSKLMNQNKMVTSELRNKLFQP 424

Query: 298 VMRI-GLDLPALNMQRSRDHGLPGYNAWRR**FCG**LPQPETVGQLGTVLRNLKLARKLMEQY 356

++ G DL A+N+QR RDHG+PGYN+WR FCGL QP+T+ L VL+N LA+KL++ Y

Sbjct: 425 THKVHGFDLAAINLQRCRDHGMPGYNSWRGFCGLSQPKTLKGLQAVLKNKVLAKKLLDLY 484

Query: 357 GTPNNIDIWMGGVSEPLKRKGRVGPLLA**C**IIG**T**QFR**K**LRDGDRFWWENEGVFSMQQRQAL 416

TP+NIDIW+GG +EP+ +GRVGPLLAC++G QF+++RDGDRFWWEN GVF+ +QR +L

Sbjct: 485 KTPDNIDIWIGGNAEPMVERGRVGPLLACLLGRQFQQIRDGDRFWWENPGVFTEKQRDSL 544

Query: 417 AQISLPRIICDNTGITTVSKNNIFMSNSYPRDFVNCSTLPALNLASW 463

++S R+ICDNT IT V + F +N+YP DFV+CS + L+L+ W

Sbjct: 545 QKVSFSRLICDNTHITKVPL-HAFQANNYPHDFVDCSAVDKLDLSPW 590

- **actual MIRs in query** (small red) = 08
- **MIRs in target** (small green)= 04
- **Common MIRs (True positive residues in query which were actually MIRs and also predicted as MIRs in alignment)** (highlighted in yellow) =00

1IA5_A

1IA5:A::**attctfsgsng**A**ss**A**sksk**T**s**C**st**I**v**LSNVAV**psg**TTL**d**LTKL**n**DG**th**V**i**FSGETTFG**yk**EWSGPLISVSGSDLTITGASGHSINGDGSRWWDGEGGNGGKTKPKFFAAHSLTNSVISGLKIVNSPVQVFSVAGSD**y**LTLKDITIDNSDGDDNGGHNTDAFDIGTSTYVTISGATVYNQDDCVAVNSGENIYFSGGYCSGGHGLSIGSVGGRSDNTVKNVTFVDSTIINSDNGVRIKTNIDTTGSVSDVTYKDITLTSIAKYGIVVQQNYGDTSSTPTTGVPITDFVLDNVHGSVVSSGTNILISCGSGSCSDWTWTDVSVSGGKTSSKCTNVPSGASC

1RMG_A

1RMG:A::QLSGSVGPLTSASTKGATKTCNILSYGAVADNSTDVGPAITSAWAACKSGGLVYIPSGNYALNTWVTLTGGSATAIQLDGII**y**RTGTASGNMIAVTDTTDFELFSSTSKGAV**q**G**f**G**y**VYHAEGTYGARILRLTDVTHFSVHDIILVDAPAFHFTMDTCSDG**e**V**y**NMAIRGGNEGGLDGIDVWGSNI**w**V**h**DVEVTNKDECVTVKSPANNILVESIYCNWSGGCAMGSLGADTDVTDIVYRNVYTWSSNQMYMIKSNGGSGTVSNVLLENFIGHGNAYSLDIDGYWSSMTAVAGDGVQLNNITVKNW**k**GTEANGATRPPIRVVCS**dt**APCTDLTLEdIAIWTESGSSE**lyl**C**r**SA**y**GSG**yc**L**k**D**s**S**s**H**tsytttstvta**AP**sg**Y**sattma**A**d**LATAF**g**L**tas**I**pi**PTI**pts**FY**pg**L**tpys**ALAG

Score = 37.4 bits (85), Expect = 5e-05, Method: Compositional matrix adjust.

Identities = 38/150 (25%), Positives = 73/150 (48%), Gaps = 8/150 (5%)

Query: 176 VYNQDDCVAVNS-GENIYFSGGYCSGGHGLSIGSVGGRSDNTVKNVTFVDSTIINSDNGV 234

V N+D+CV V S NI YC+ G ++GS+G +D V ++ + + +S+

Sbjct: 193 VTNKDECVTVKSPANNILVESIYCNWSGGCAMGSLGADTD--VTDIVYRNVYTWSSNQMY 250

Query: 235 RIKTNIDTTGSVSDVTYKDITLTSIAKYGIVVQQNYGDTSSTPTTGVPITDFVLDNVHGS 294

IK+N +G+VS+V ++ A Y + + + ++ GV + + + N G+

Sbjct: 251 MIKSN-GGSGTVSNVLLENFIGHGNA-YSLDIDGYWSSMTAVAGDGVQLNNITVKNW**K**GT 308

Query: 295 VVSSGTN--ILISCG-SGSCSDWTWTDVSV 321

+ T I + C + C+D T D+++

Sbjct: 309 EANGATRPPIRVVCS**DT**APCTDLTLEDIAI 338

- **actual MIRs in query** (small red) = 32
- **MIRs in target** (small green)= 57
- **Common MIRs (True positive residues in query which were actually MIRs and also predicted as MIRs in alignment)** (highlighted in yellow) =00

1Q8O_A

1Q8O:A::EDSLSFGFPTFPSDQKNLIFQGDAQIKNNAVQLTKTDSNGNPVA**s**T**v**GRILFSAQVHLWEKSSSRVANFQSQFSFSLKSPLSNG**ad**GIAFFIAPPDTTIPS**g**S**gggl**LGLFAPGTAQNTSANQVIAVEFDT**f**YAQ**dsn**T**w**DPNYPHIGIDVNSIRSVKTVKWDRRDGQSLNVLVTFNPSTRNLDVVATYSDGTRYEVSYEVDVRSVLPEWVRVGFSAAS**geq**YQTHTLESWSFTSTLLYTAQKKGENLALEM

1BQP_A

1BQP:A::TETTSFLITKFSPDQQNLIFQGDGYTTKEKLTLTKAVK**n**TVGRALYSSPIHIWDRETGNVANFVTSFTFVINAPNSYNV**ad**GFTFFIAPVDTKPQTG**ggy**LGVFNSAEYDKTTQTVAVEFDT**f**Y**n**AA**w**DPSNRDRHIGIDVNSIKSVNTKSWKLQNGEEANVVIAFNAATNVLTVSLTYPN

Score = 145 bits (365), Expect = 1e-37, Method: Compositional matrix adjust.

Identities = 85/191 (44%), Positives = 115/191 (60%), Gaps = 16/191 (8%)

Query: 2 DSLSFGFPTFPSDQKNLIFQGDAQIKNNAVQLTKTDSNGNPVA**S**T**V**GRILFSAQVHLWEK 61

++ SF F DQ+NLIFQGD + LTK V +TVGR L+S+ +H+W++

Sbjct: 2 ETTSFLITKFSPDQQNLIFQGDGYTTKEKLTLTK------AVK**N**TVGRALYSSPIHIWDR 55

Query: 62 SSSRVANFQSQFSFSLKSPLS-NG**AD**GIAFFIAPPDTTIPS**G**S**GGGL**LGLFAPGTAQNTS 120

+ VANF + F+F + +P S N ADG FFIAP DT +GGG LG+F +A+

Sbjct: 56 ETGNVANFVTSFTFVINAPNSYNV**AD**GFTFFIAPVDT--KPQTG**GGY**LGVF--NSAEYDK 111

Query: 121 ANQVIAVEFDT**F**YAQ**DSN**T**W**DPNYP--HIGIDVNSIRSVKTVKWDRRDGQSLNVLVTFNP 178

Q +AVEFDTFY + WDP+ HIGIDVNSI+SV T W ++G+ NV++ FN

Sbjct: 112 TTQTVAVEFDT**F**Y---**N**AAWDPSNRDRHIGIDVNSIKSVNTKSWKLQNGEEANVVIAFNA 168

Query: 179 STRNLDVVATY 189

+T L V TY

Sbjct: 169 ATNVLTVSLTY 179

- **actual MIRs in query** (small red) = 17
- **MIRs in target** (small green)= 09
- **Common MIRs (True positive residues in query which were actually MIRs and also predicted as MIRs in alignment)** (highlighted in yellow) =09

2E22_A

2E22:A::SDEFDALRIKWATLLTGGPALDPADSDIAARTDKLAQDANDYWEDMDLSSSRTYIWYALRGNGTSDNVNAVYERLRTMALAATTVGSSLYGNADLKEDILDALDWLYVNSYNSTRSRSAY**n**W**wh**WQLGIPMSLNDIAVLLYDDISAARMATYMDTIDYFTPSIGLTGA**n**RA**w**QAIVVGVRAVIVKDAVKLAAARNGLSGTGIFPYATGGDGFYADGSFVQHTTFAYTGG**y**GS**s**VLETTANLMYLLSGSTWSVSDPNQSNVWQWIYEAYRPLLYKGAMMDMVRG**re**IS**r**S**y**AQDHAVGHGIVASIVRLAQFAPAPHAAAFKQIAKRVIQEDTFSSFYGDVSTDTIRLAKAIVDDPSIAPAAAPNLYKQYAAMDRAVLQRPGFALGLALYSTRISSYESI**n**SENGRGWYTGAGATYLYNQDLAQYSEDYWPTVDAYRIPGTTVASGTPIASGTGTSSWTGGVSLAGQYGASGMDLSYGAYNLSARKSWFMFDDEIVALGSGISSTAGIPIETVVDNRKLNGAGDNAWTANGAALSTGLGVAQTLTGVNWVHLAGNTADGSDIGYYFPGGATLQTKREARTGTWKQINN**r**PATPSTAVTRNYETMWIDHGTNPSGASYGYVLLPNKTSAQVGAYAADPAIEIVVNTSGVQSVKEKTLGLVGANFWTDTTQTADLITSNKKASVMTREIADERLEASVSDPTQANNGTIAIELARSAEGYSADPGITVTQLAPTIKFTVNVNGAKGKSFHASFQLG

1HM2_A

1HM2:A::MKKLFVTCIVFFSILSPALLIAQQTGTAELIMKRVMLDLKKPLRNMDKVAEKNLNTLQPDGSWKDVPYKDDAMTNWLPNNHLLQLETIIQAYIEKDSHYYGDDKVFDQISKAFKYWYDSDPKSRNWWHNEIATPQALGEMLILMRYGKKPLDEALVHKLTERMKRGEPEKKTGANKTDIALHYFYRALLTSDEALLSFAVKELFYPVQFVHYEEGLQYDYSYLQHGPQLQISSYGAVFITGVLKLANYVRDTPYALSTEKLAIFSKYYRDSYLKAIRGSYMDFNVEGRGVSRPDILNKKAEKKRLLVAKMIDLKHTEEWADAIARTD**stva**AGYKIEPYHHQFWNGDYVQHLRPAYSFNVRMVSKRTRRSESGNKENLLGRYLSDGATNIQLRGPEYYNIMPVWEWDKIPGITS**r**DYLTDRPLTKLWGEQGSNDFAGGVSDGVYGASAYALDYD**s**LQAKKAWFFFDKEIVCLGAGINS**nap**ENITTTLNQSWLNGPVISTAGKTGRGKITTFKAQGQFWLLHDAIGYYFPEGANLSLSTQSQKGNWFHINNSHSKDEVSGDVFKLWINHGARPENAQYAYIVLPGINKPEEIKKYNGTAPKVLANTNQLQAVYHQQLDMVQAIFYTAGKLSVAGIEIETDKPCAVLIKHINGKQVIWAADPLQKEKTAVLSIRDLKTGKTNRVKIDFPQQEFAGATVELK

Score = 147 bits (371), Expect = 9e-38, Method: Compositional matrix adjust.

Identities = 163/646 (25%), Positives = 267/646 (41%), Gaps = 74/646 (11%)

Query: 74 RLRTMALAATTVGSSLYGNADLKEDILDALDWLYVNSYNSTRSRSAY**N**W**WH**WQLGIPMSL 133

+L T+ A S YG+ + + I A + Y + +SR NWWH ++ P +L

Sbjct: 84 QLETIIQAYIEKDSHYYGDDKVFDQISKAFKYWYDSD---PKSR---NWWHNEIATPQAL 137

Query: 134 NDIAVLLY---DDISAARMATYMDTIDYFTPSIGLTGA**N**RA**W**QAIVVGVRAVIVKDAVKL 190

++ +L+ + A + + + P TGAN+ A+ RA++ D L

Sbjct: 138 GEMLILMRYGKKPLDEALVHKLTERMKRGEPE-KKTGANKTDIALHYFYRALLTSDEALL 196

Query: 191 AAARNGLSGTGIFP--YATGGDGFYADGSFVQHTTFAYTGG**Y**GS**S**VLETTANLMYLLSGS 248

+ A L +P + +G D S++QH YG+ + L + +

Sbjct: 197 SFAVKEL----FYPVQFVHYEEGLQYDYSYLQHGPQLQISSYGAVFITGVLKLANYVRDT 252

Query: 249 TWSVSDPNQSNVWQWIYEAYRPLLYKGAMMDM-VRG**RE**IS**R**S**Y**AQDHAVGHGIVASIVRL 307

+++S + ++ ++Y + +G+ MD V GR +SR

Sbjct: 253 PYALSTEKLAIFSKYYRDSYLKAI-RGSYMDFNVEGRGVSR------------------- 292

Query: 308 AQFAPAPHAAAFKQIAKRVIQEDTFSSFYGDVSTDTIRLAKAIVDDPSIAPAAAPNLYKQ 367

P K KR++ + + D I A D A + Q

Sbjct: 293 ------PDILNKKAEKKRLLVAKMIDLKHTEEWADAI----ARTD**STVA**AGYKIEPYHHQ 342

Query: 368 YAAMDRAVLQRPGFALGLALYSTRISSYESI**N**SENGRGWYTGAGATYLYNQDLAQYSEDY 427

+ D RP ++ + + S R ES N EN G Y GAT + + Y +

Sbjct: 343 FWNGDYVQHLRPAYSFNVRMVSKRTRRSESGNKENLLGRYLSDGATNIQLRGPEYY--NI 400

Query: 428 WPTVDAYRIPGTT---VASGTPIAS---GTGTSSWTGGVSLAGQYGASGMDLSYGAYNLS 481

P + +IPG T + P+ G++ + GGVS G YGAS L Y + L

Sbjct: 401 MPVWEWDKIPGITS**R**DYLTDRPLTKLWGEQGSNDFAGGVS-DGVYGASAYALDYD**S**--LQ 457

Query: 482 ARKSWFMFDDEIVALGSGISSTAGIPIETVVDNRKLNGAGDNAWTANGAALSTGLGVAQT 541

A+K+WF FD EIV LG+GI+S A I T ++ LNG A TG G T

Sbjct: 458 AKKAWFFFDKEIVCLGAGINS**NAP**ENITTTLNQSWLNGP------VISTAGKTGRGKITT 511

Query: 542 LTGVNWVHLAGNTADGSDIGYYFPGGATLQTKREARTGTWKQINN**R**PATPSTAVTRNYET 601

L + IGYYFP GA L +++ G W INN + V+ +

Sbjct: 512 FKAQGQFWLLHDA-----IGYYFPEGANLSLSTQSQKGNWFHINN--SHSKDEVSGDVFK 564

Query: 602 MWIDHGTNPSGASYGYVLLPNKTSAQVGAYAADPAIEIVVNTSGVQSVKEKTLGLVGANF 661

+WI+HG P A Y Y++LP + A +++ NT+ +Q+V + L +V A F

Sbjct: 565 LWINHGARPENAQYAYIVLPGINKPEEIKKYNGTAPKVLANTNQLQAVYHQQLDMVQAIF 624

Query: 662 WT--DTTQTADLITSNKKASVMTREIADERLEASVSDPTQANNGTI 705

+T + I ++K +V+ + I +++ +DP Q +

Sbjct: 625 YTAGKLSVAGIEIETDKPCAVLIKHINGKQV-IWAADPLQKEKTAV 669

- **actual MIRs in query** (small red) = 13
- **MIRs in target** (small green)= 09
- **Common MIRs (True positive residues in query which were actually MIRs and also predicted as MIRs in alignment)** (highlighted in yellow) =00

2OK5_A

2OK5:A::GSHHHHHHGSTPWSLARPQGSCSLEGVEI**k**GGSFRLLQEGQALEYVCPSGFYPYPVQTRTCRSTGSWSTLKTQDQKTVRKA**e**CrAIHCPRPHDFENGEYWPRSPYYNVSDEISFHCYDGYTLRGSANRTCQVNGRWSGQTAICDNGAGYCSNPGIPIGTRKVGSQYRLEDSVTYHCSRGLTLRGSQRRTCQEGGSWSGTEPSCQDSFMYDTPQEVAEAFLSSLTETIEGVDAEDGHGPGEQQKRKIVLDPSGSMNIYLVLDGSDSIGASNFTGAKKCLVNLIEKVASYGVKPRYGLVTYATYPKIWVKVSEADSSNADWVTKQLNEINYEDHKLKSGTNTKKALQAVYSMMSWPDDVPPEGWNRTRHVIILMTDGLHNMGGDPITVIDEIRDLLYIGKDRKNPREDYLDVYVFGVGPLVNQVNINALASKKDNEQHVFKVKDMENLEDVFYQMIDESQSLSLCGMVWEHRKGTDYHKQPWQAKISVIRPSKGHESCMGAVVSEYFVLTAAHCFTVDDKEHSIKVSVGGEKRDLEIEVVLFHPNYNINGKKEAGIPEFYDYDVALIKLKNKLKYGQTIRPICLPCTEGTTRALRLPPTTTCQQQKEELLPAQDIKALFVSEEEKKLTRKEVYIKNGDKKGSCERDAQYAPGYDKVKDISEVVTPRFLCTGGVSPYADPNTCRGDSGGPLIVHKRSRFIQVGVISWGVVDVCKNQKRQ**k**QVPAHARDFHINLFQVLPWLKEKLQDEDLGFLAAA

>1GPZ_A

1GPZ:A::IKCPQPKTLDEFTIIQNL**q**PQYQFRDYFIATCKQGYQLIEGNQVLHSFTAVCQDDGTWHRAMPRCKIKDCGQPRNLPNGDFRYTTTMGVNTYKARIQYYCHEPYYKMQTRAGSRESEQGVYTCTAQGIWKNEQKGEKIPRCLPVCGKPVNPVEQRQQIIGGQKAKMGNFPWQVFTNIHGRGGGALLGDRWILTAAHTLYPKEHEAQSNASLDVFLGHTNVEELMKLGNHPIRRVSVHPDYRQDESYNFEGDIALLELENSVTLGPNLLPICLPDNDTFYDLGLMGYVSGFGVMEEKIAHDLRFVRLPVANPQACENWLRGKNRMDVFSQNMFCAGHPSLKQDACQGDSGGVFAVRDPNTDRWVATGIVSWGIGCSRGYGFYTKVLNYVDWIKKEMEEED

Score = 60.1 bits (144), Expect = 2e-11, Method: Compositional matrix adjust.

Identities = 66/277 (23%), Positives = 121/277 (43%), Gaps = 57/277 (20%)

Query: 479 PWQAKISVIRPSKGHESCMGAVVSEYFVLTAAHCFTVDDKEH------SIKVSVGGEKRD 532

PWQ ++ H GA++ + ++LTAAH T+ KEH S+ V +G +

Sbjct: 170 PWQVFTNI------HGRGGGALLGDRWILTAAH--TLYPKEHEAQSNASLDVFLGHTNVE 221

Query: 533 LEIEVVLFHPNYNINGKKEAGIPEFYDY--DVALIKLKNKLKYGQTIRPICLPCTEGTTR 590

E+ + HP ++ + E Y++ D+AL++L+N + G + PICLP + T

Sbjct: 222 -ELMKLGNHPIRRVSVHPDYRQDESYNFEGDIALLELENSVTLGPNLLPICLPDND-TFY 279

Query: 591 ALRLPPTTTCQQQKEELLPAQDIKALFVSEEEKKLTRKEVYIKNGDKKGSCERDAQYAPG 650

L L + EE + A D++ + + + +CE

Sbjct: 280 DLGLMGYVSGFGVMEEKI-AHDLRFVRLPVANPQ---------------ACEN------- 316

Query: 651 YDKVKDISEVVTPRFLCTGGVSPYADPNTCRGDSGGPLIVH--KRSRFIQVGVISWGVVD 708

+ + K+ +V + C G P + C+GDSGG V R++ G++SWG+

Sbjct: 317 WLRGKNRMDVFSQNMFCAG--HPSLKQDACQGDSGGVFAVRDPNTDRWVATGIVSWGI-- 372

Query: 709 VCKNQKRQ**K**QVPAHARDFHINLFQVLPWLKEKLQDED 745

C + F+ + + W+K+++++ED

Sbjct: 373 GC----------SRGYGFYTKVLNYVDWIKKEMEEED 399

- **actual MIRs in query** (small red) = 04
- **MIRs in target** (small green)= 01
- **Common MIRs (True positive residues in query which were actually MIRs and also predicted as MIRs in alignment)** (highlighted in yellow) =00

1GPE_A

1GPE:A::YLPAQQIDVQSSLLSDPSKVAGKTYDYIIAGGGLTGLTVAAKLTENPKIKVLVIEKGFYESNDGAIIEDPNAYGQIFGTTVDQNYLTVPLINNRTNNIKAGKGLGGSTLINGDSWTRPDKVQIDSWEKVFGMEGWNWDNMFEYMKKAEAARTPTAAQLAAGHSFNATCHGTNGTVQSGARDNGQPWSPIMKALMNTVSALGVPVQQDFLCGHPRGVSMIMNNLDENQVRVDAARAWLLPNYQRSNLEILTGQMVGKVLFKQTASGPQAVGVNFGTNKAVNFDVFAKHEVLLAAGSAISPLILEYSGIGLKSVLDQANVTQLLDLPVGINMQDQTTTTVSS**r**ASSAGAGQGQAVFFANFTETFGDYAPQARDLLNTKLDQWAEETVARGGFHNVTALKVQYENYRNWLLDEDVAFAELFMDTEGKINFDLWDLIPFTRGSVHILSSDPYLWQFANDPKFFLNEFDLLGQAAASKLARDLTSQGAMKEY**f**A**ge**TLP**gy**NLV**q**NATLSQWSDYVLQNFRPNWHAVSSCSMMSRELGGVVDATAKVYGTQGLRVIDGSIPPTQVSSHVMTIFYGMALKVADAILDDYAKSA

1JU2_A

1JU2:A::LATTSDHDFSYLSFAYDATDLELEGSYDYVIVGGGTSGCPLAATLSEKYKVLVLERGSLPTAYPNVLTADGFVYNLQQEDDGKTPVERFVSEDGIDNVRGRVLGGTSIINAGVYARANTSIYSASGVDWDMDLVNQTYEWVEDTIVYKPNSQSWQSVTKTAFLEAGVH**p**N**h**GF**s**LDH**e**EGTRITGSTFDNKGTRHAADELLNKGNSNNLRVGVHASVEKIIFSNAPGLTATGVIYRDSNGTPHQAFVRSKGEVIVSAGTIGTPQLLLLSGVGPESYLSSLNIPVVLSHPYVGQFLHDNPRNFINILPPNPIEPTIVTVLGISNDFYQCSFSSLPFTTPPFGFFPSSSYPLPNSTFAHFASKVAGPLSYGSLTLKSSSNVRVSPNVKFNYYSNLTDLSHCVSGMKKIGELLSTDALKPYKVEDLPGVEGFNILGIPLPKDQTDDAAFETFCRESVASYWHYHGGCLVGKVLDGDFRVTGINALRVVDGSTFPYTPASHPQGFYLMLGRYVGIKILQERSASDLKILDSLKSAASLVL

Score = 73.9 bits (180), Expect = 1e-15, Method: Compositional matrix adjust.

Identities = 136/596 (22%), Positives = 218/596 (36%), Gaps = 134/596 (22%)

Query: 24 TYDYIIAGGGLTGLTVAAKLTENPKIKVLVIEKGFYESNDGAIIEDPNAYGQIF------ 77

+YDY+I GGG +G +AA L+E K KVLV+E+G P AY +

Sbjct: 26 SYDYVIVGGGTSGCPLAATLSE--KYKVLVLERG----------SLPTAYPNVLTADGFV 73

Query: 78 --------GTTVDQNYLTVPLINNRTNNIKAGKGLGGSTLINGDSWTRPDKVQIDSWEKV 129

G T + +++ + +N++ G+ LGG+++IN + R + +

Sbjct: 74 YNLQQEDDGKTPVERFVS----EDGIDNVR-GRVLGGTSIINAGVYARANT-------SI 121

Query: 130 FGMEGWNWDNMFEYMKKAEAARTPTAAQLAAGHSFNATCHGTNGTVQSGARDNGQPWSPI 189

+ G +WD N T T+ + N Q W +

Sbjct: 122 YSASGVDWDMDL----------------------VNQTYEWVEDTIV--YKPNSQSWQSV 157

Query: 190 MKALMNTVSALGVPVQQDFLCGHPRGVSMIMNNLDENQVRVDAARAWLLPNYQRSNLEIL 249

K GV F H G + + D R A LL +NL +

Sbjct: 158 TKTAFLEA---GVH**P**N**H**GF**S**LDH**E**EGTRITGSTFDNKGTR--HAADELLNKGNSNNLRVG 212

Query: 250 TGQMVGKVLFKQTASGPQAVGVNFGTNKAVNFDVF--AKHEVLLAAGSAISPLILEYSGI 307

V K++F A G A GV + + F +K EV+++AG+ +P +L SG+

Sbjct: 213 VHASVEKIIFSN-APGLTATGVIYRDSNGTPHQAFVRSKGEVIVSAGTIGTPQLLLLSGV 271

Query: 308 GLKSVLDQANVTQLLDLP-VGINMQDQT-------------TTTVSS**R**ASSAGAGQGQAV 353

G +S L N+ +L P VG + D T V+ S Q

Sbjct: 272 GPESYLSSLNIPVVLSHPYVGQFLHDNPRNFINILPPNPIEPTIVTVLGISNDFYQCSFS 331

Query: 354 FFANFTETFGDYAPQARDLLNTKLDQWAEETVARGGFHNVTALKVQYENYRNWLLDEDVA 413

T FG + + L N+ +A + + ++T ++ N + +V

Sbjct: 332 SLPFTTPPFGFFPSSSYPLPNSTFAHFASKVAGPLSYGSLT-----LKSSSNVRVSPNVK 386

Query: 414 FAELFMDTEGKINFDLWDLIPFTRGSVHILSSD---PYLWQFANDPKFFLNEFDLLGQAA 470

F T DL + + +LS+D PY + + P + F++LG

Sbjct: 387 FNYYSNLT------DLSHCVSGMKKIGELLSTDALKPY--KVEDLPG--VEGFNILGI-- 434

Query: 471 ASKLARDLTSQGAMKEY**F**A**GE**TLP**GY**NLV**Q**NATLSQWSDYVLQNFRPNWHAVSSCSMMSR 530

L +D T A E F E++ Y WH C +

Sbjct: 435 --PLPKDQTDDAAF-ETFCRESVASY----------------------WHYHGGCLV--- 466

Query: 531 ELGGVVDATAKVYGTQGLRVIDGSIPPTQVSSHVMTIFYGMALKVADAILDDYAKS 586

G V+D +V G LRV+DGS P +SH + + V IL + + S

Sbjct: 467 --GKVLDGDFRVTGINALRVVDGSTFPYTPASHPQGFYLMLGRYVGIKILQERSAS 520

- **actual MIRs in query** (small red) = 07
- **MIRs in target** (small green)= 04
- **Common MIRs (True positive residues in query which were actually MIRs and also predicted as MIRs in alignment)** (highlighted in yellow) =00

1KDG_A

1KDG:A::PTVSA**t**P**y**DYIIVGAGPGGIIAADRLSEAGK**k**VLLLERGGPSTKQTGGTYVAPWATSSGLTKFDIPGLFESLFTDSNPFWWCKDITVFAGCLVGGGTSVNGALYWYPNDGDFSSSVGWPSSWTNHAPYTSKLSSRLPSTDHPSTDGQRYLEQSFNVVSQLLKGQGYNQATINDNPNYKDHVFGYSAFDFLNGKRAGPVATYLQTALARPNF**t**FKTNVMVSNVVRNGSQILGVQTNDPTLGPNGFIPVTPKGRVILSAGAFGTSRILFQSGIGPTDMIQTVQSNPTAAAALPPQNQWINLPVGMNAQDNPSINLVFTHPSIDAYENWADVWSNPRPADAAQYLANQSGVFAGASPKLNFWRAYSGSDGFTRYAQGTVRPGAASVNSSLPYNASQIFTITVYLSTGIQSRGRIGIDAALRGTVLTPPWLVNPVDKTVLLQALHDVVSNIGSIPGLTMITPDVTQTLEEYVDAYDPATMNSNHWVSSTTIGSSPQSAVVDSNVKVFGTNNLFIVDAGIIPHLPTGNPQGTLMSAAEQAAAKILALAGGP

1GPE_A

1GPE:A::YLPAQQIDVQSSLLSDPSKVAGKTYDYIIAGGGLTGLTVAAKLTENPKIKVLVIEKGFYESNDGAIIEDPNAYGQIFGTTVDQNYLTVPLINNRTNNIKAGKGLGGSTLINGDSWTRPDKVQIDSWEKVFGMEGWNWDNMFEYMKKAEAARTPTAAQLAAGHSFNATCHGTNGTVQSGARDNGQPWSPIMKALMNTVSALGVPVQQDFLCGHPRGVSMIMNNLDENQVRVDAARAWLLPNYQRSNLEILTGQMVGKVLFKQTASGPQAVGVNFGTNKAVNFDVFAKHEVLLAAGSAISPLILEYSGIGLKSVLDQANVTQLLDLPVGINMQDQTTTTVSS**r**ASSAGAGQGQAVFFANFTETFGDYAPQARDLLNTKLDQWAEETVARGGFHNVTALKVQYENYRNWLLDEDVAFAELFMDTEGKINFDLWDLIPFTRGSVHILSSDPYLWQFANDPKFFLNEFDLLGQAAASKLARDLTSQGAMKEY**f**A**ge**TLP**gy**NLV**q**NATLSQWSDYVLQNFRPNWHAVSSCSMMSRELGGVVDATAKVYGTQGLRVIDGSIPPTQVSSHVMTIFYGMALKVADAILDDYAKSA

Score = 53.5 bits (127), Expect = 1e-09, Method: Compositional matrix adjust.

Identities = 147/621 (23%), Positives = 224/621 (36%), Gaps = 143/621 (23%)

Query: 3 VSA**T**P**Y**DYIIVGAGPGGIIAADRLSEAGK-**K**VLLLERGGPSTKQTGGTYVAPWATSSGLT 61

V+ YDYII G G G+ A +L+E K KVL++E+G + S+

Sbjct: 20 VAGKTYDYIIAGGGLTGLTVAAKLTENPKIKVLVIEKG--------------FYESNDGA 65

Query: 62 KFDIPGLFESLF---TDSN----PFWWCKDITVFAGCLVGGGTSVNGALYWYPNDGDFSS 114

+ P + +F D N P + + AG +GG T +NG + P+ S

Sbjct: 66 IIEDPNAYGQIFGTTVDQNYLTVPLINNRTNNIKAGKGLGGSTLINGDSWTRPDKVQIDS 125

Query: 115 ------SVGWPSSWTNHAPYTSKL-SSRLPSTDHPSTDGQRYLEQSFNVVSQLLKGQGYN 167

GW +W N Y K ++R P T Q SFN G

Sbjct: 126 WEKVFGMEGW--NWDNMFEYMKKAEAARTP------TAAQLAAGHSFNATCH--GTNGTV 175

Query: 168 QATINDN-----PNYKDHVFGYSAF------DFLNGK----------------RAGPVAT 200

Q+ DN P K + SA DFL G R

Sbjct: 176 QSGARDNGQPWSPIMKALMNTVSALGVPVQQDFLCGHPRGVSMIMNNLDENQVRVDAARA 235

Query: 201 YLQTALARPNF**T**FKTNVMVSNVV----RNGSQILGVQTNDPTLGPNGFI--PVTPKGRVI 254

+L R N T MV V+ +G Q +GV G N + V K V+

Sbjct: 236 WLLPNYQRSNLEILTGQMVGKVLFKQTASGPQAVGV-----NFGTNKAVNFDVFAKHEVL 290

Query: 255 LSAGAFGTSRILFQSGIGPTDMIQTVQSNPTAAAALPPQNQWINLPVGMNAQDNPSINLV 314

L+AG+ + IL SGIG ++ Q+N T Q ++LPVG+N QD + +

Sbjct: 291 LAAGSAISPLILEYSGIGLKSVLD--QANVT---------QLLDLPVGINMQDQTTTTVS 339

Query: 315 FTHPSIDAYENWADVWSNPRPADAAQYLANQSGVFAGASPKLNFWRAYSGSDGFTRYAQG 374

S A + A + AN + F +P+ R + ++A+

Sbjct: 340 S**R**ASSAGAGQG------------QAVFFANFTETFGDYAPQA---RDLLNTK-LDQWAEE 383

Query: 375 TVRPGAASVNSSLP-----YNASQIFTITVYLSTGIQSRGRIGIDA-----ALRGTVLT- 423

TV G ++L Y + + + + G+I D RG+V

Sbjct: 384 TVARGGFHNVTALKVQYENYRNWLLDEDVAFAELFMDTEGKINFDLWDLIPFTRGSVHIL 443

Query: 424 ------------PPWLVNPVD---KTVLLQALHDVVSNIG--------SIPGLTMITPDV 460

P + +N D + + D+ S ++PG ++

Sbjct: 444 SSDPYLWQFANDPKFFLNEFDLLGQAAASKLARDLTSQGAMKEY**F**A**GE**TLP**GY**NLV**Q**--- 500

Query: 461 TQTLEEYVDAYDPATMNSN-HWVSSTTIGSSPQSAVVDSNVKVFGTNNLFIVDAGIIPHL 519

TL ++ D Y N H VSS ++ S VVD+ KV+GT L ++D I P

Sbjct: 501 NATLSQWSD-YVLQNFRPNWHAVSSCSMMSRELGGVVDATAKVYGTQGLRVIDGSIPPTQ 559

Query: 520 PTGNPQGTLMSAAEQAAAKIL 540

+ + A + A IL

Sbjct: 560 VSSHVMTIFYGMALKVADAIL 580

- **actual MIRs in query** (small red) = 04
- **MIRs in target** (small green)= 07
- **Common MIRs (True positive residues in query which were actually MIRs and also predicted as MIRs in alignment)** (highlighted in yellow) =00

1O0V_A

1O0V:A::DIVASRDFTPPTVKILQSSCDGGGHFPPTIQLLCLVSGYTPGTIQITWLEDGQVMDVDLSTASTTQEGELASTQSELTLSQKHWLSDRTYTCQVTYQGHTFEDSTKKCADSNPRGVS**a**Y**l**S**rp**SP**f**DLFIRKS**p**T**i**T**c**LVVDLAPSKGTVQLTWSRASGKPVNHSTRKEEKQRNGTLT**v**T**s**TLPVGTRDWIEGETYQCRVTHPHLPRALMRSTTKTSGPRAAPEVYAFATPEWPGSRDKRTLACLIQNFMPEDISVQWLHNEVQLPDARHST**t**QPRKTKGSGFFVFSRLEVTRAEWEQKDEFICRAVHEAASPSQTVQRAVSVNPGL

1HZH_H

1HZH:H::QVQLVQSGAEVKKPGASVKVSCQASGYRFSNFVIHWVRQAPGQRFEWMGWINPYNGNKEFSAKFQDRVTFTADTSANTAYMELRSLRSADTAVYYCARVGPYSWDDSPQDNYYMDVWGKGTTVIVSSASTKGPSVFPLAPSSKSTSGGTAALGCLVKDYFPEPVTVSWNSGALTSGVHTFPAVLQSSGLYSLSSVVTVPSSSLGTQTYICNVNHKPSNTKVDKKAEPKSCDKTHTCPPCPAPELLGGPSVFLF**p**P**k**PKDTLMISRTPEVTCVVVDVSHEDPEVKFNWYVDGVEVHNAKTKPREEQYNSTYRVVSVLTVLHQDWLNGKEYKCKVSNKALPAPIEKTISKAKGQ**p**REPQVYTLPPSRDELTKNQVSLTCLVKGFYPSDIAVEWESNGQPENNYKTTPPVLDSDGSFFLYSKLTVDKSRWQQGNVFSCSVMHEALHNHYTQKSLSLSPGK

Score = 135 bits (340), Expect = 2e-34, Method: Compositional matrix adjust.

Identities = 104/347 (29%), Positives = 159/347 (45%), Gaps = 36/347 (10%)

Query: 2 IVASRDFTPPTVKIL--QSSCDGGGHFPPTIQLLCLVSGYTPGTIQITWLEDGQVMDVDL 59

IV+S P+V L S GG T L CLV Y P + ++W V

Sbjct: 124 IVSSASTKGPSVFPLAPSSKSTSGG----TAALGCLVKDYFPEPVTVSWNSGALTSGVHT 179

Query: 60 STASTTQEGELASTQSELTLSQKHWLSDRTYTCQVTYQGHTFE----------DSTKKC- 108

A G + + S L +TY C V ++ + D T C

Sbjct: 180 FPAVLQSSGLYSLSSVVTVPSSS--LGTQTYICNVNHKPSNTKVDKKAEPKSCDKTHTCP 237

Query: 109 ---ADSNPRGVS**A**Y**L**S**RP**SP**F**D-LFIRKS**P**T**I**T**C**LVVDLAPSKGTVQLTWSRASGKPVNH 164

A G S +L P P D L I ++P +TC+VVD++ V+ W + N

Sbjct: 238 PCPAPELLGGPSVFLF**P**P**K**PKDTLMISRTPEVTCVVVDVSHEDPEVKFNWYVDGVEVHNA 297

Query: 165 STRKEEKQRNGTLT**V**T**S**TLPVGTRDWIEGETYQCRVTHPHLPRALMRSTTKTSGPRAAPE 224

T+ E+Q N T V S L V +DW+ G+ Y+C+V++ LP + ++ +K G P+

Sbjct: 298 KTKPREEQYNSTYRVVSVLTVLHQDWLNGKEYKCKVSNKALPAPIEKTISKAKGQ**P**REPQ 357

Query: 225 VYAFATPEWPGSRDKRT-----LACLIQNFMPEDISVQWLHNEVQLPDARHST**T**QPRKTK 279

VY P SRD+ T L CL++ F P DI+V+W N P+ + TT P

Sbjct: 358 VYTL-----PPSRDELTKNQVSLTCLVKGFYPSDIAVEWESN--GQPENNYKTTPPVLDS 410

Query: 280 GSGFFVFSRLEVTRAEWEQKDEFICRAVHEAASPSQTVQRAVSVNPG 326

FF++S+L V ++ W+Q + F C +HEA + Q+++S++PG

Sbjct: 411 DGSFFLYSKLTVDKSRWQQGNVFSCSVMHEALH-NHYTQKSLSLSPG 456

- **actual MIRs in query** (small red) = 11
- **MIRs in target** (small green)= 03
- **Common MIRs (True positive residues in query which were actually MIRs and also predicted as MIRs in alignment)** (highlighted in yellow) =01

1QMO_A

1QMO:A::AQSLSFSFTKFDPNQEDLIFQGHATSTNNVLQVTKLDSAGNPVSSSAGRVLYSAPLRLWEDSAVLTSFDTIINFEISTPYTSRI**ad**GLAFFIAPPDSVISY**hg**G**f**LGLFPNAN

1Q8O_A

1Q8O:A::EDSLSFGFPTFPSDQKNLIFQGDAQIKNNAVQLTKTDSNGNPVA**s**T**v**GRILFSAQVHLWEKSSSRVANFQSQFSFSLKSPLSNG**ad**GIAFFIAPPDTTIPS**g**S**gggl**LGLFAPGTAQNTSANQVIAVEFDT**f**YAQ**dsn**T**w**DPNYPHIGIDVNSIRSVKTVKWDRRDGQSLNVLVTFNPSTRNLDVVATYSDGTRYEVSYEVDVRSVLPEWVRVGFSAAS**geq**YQTHTLESWSFTSTLLYTAQKKGENLALEM

Score = 95.1 bits (235), Expect = 5e-23, Method: Compositional matrix adjust.

Identities = 50/98 (51%), Positives = 69/98 (70%), Gaps = 2/98 (2%)

Query: 3 SLSFSFTKFDPNQEDLIFQGHATSTNNVLQVTKLDSAGNPVSSSAGRVLYSAPLRLWED- 61

SLSF F F +Q++LIFQG A NN +Q+TK DS GNPV+S+ GR+L+SA + LWE

Sbjct: 3 SLSFGFPTFPSDQKNLIFQGDAQIKNNAVQLTKTDSNGNPVA**S**T**V**GRILFSAQVHLWEKS 62

Query: 62 SAVLTSFDTIINFEISTPYTSRI**AD**GLAFFIAPPDSVI 99

S+ + +F + +F + +P S ADG+AFFIAPPD+ I

Sbjct: 63 SSRVANFQSQFSFSLKSPL-SNG**AD**GIAFFIAPPDTTI 99

- **actual MIRs in query** (small red) = 05
- **MIRs in target** (small green)= 17
- **Common MIRs (True positive residues in query which were actually MIRs and also predicted as MIRs in alignment)** (highlighted in yellow) =02

1R46_A

1R46:A::L**d**N**g**LA**r**TPTMGWLHWERFMCNLDCQEEPDSCISEKLFMEMAELMVSEGWKDAGYEYLCIDDCWMAPQRDSEGRLQADPQRFPHGIRQLANYVHSKGLKLGIYADVGNKTCAGFPGSFGYYDIDAQTFADWGVDLLKFDGCYCDSLENLADGYKHMSLALNRTGRSIVYSCEWPLYMWPFQKPNYTEIRQYCNHWRNFADIDDSWKSIKSILDWTSFNQERIVDVAG**p**GGWNDPDMLVIGNFGLSWNQQVTQMALWAIMAAPLFMSNDLRHISPQAKALLQDKDVIAINQDPLGKQGYQLRQGDNFEVWERPLSGLAWAVAMINRQEIGGPRSYTIAVASLGKGVACNPACFITQLLPVKRKLGFYEWTSRLRSHINPTGTVLLQLENTMQMSLKDLL

1SZN_A

1SZN:A::IVMPDGVTG**k**VPSLGWNSWNAYHCDIDESKFLSAAELIVSSGLLDAGYNYVNIDDCWSMKDGRVDGHIAPNATRFPDGIDGLAKKVHALGLKLGIYSTAGTATCAGYPASLGYEDVDAADFADWGVDYLKYDNCNVPSDWQDEYVACNPDFVKTGPNGTCTTALDPTLAPPGYDWSTSKSAERFGAMRNALAKQSHEIVLSMCIWGQADVFSWGNSTGISWRMSDDISPNWGSVTRILNLNS**f**K**ln**SVD**f**WGHNDADMLEVGNGNLTAAETRTHFALWAAMKSPLLIGTDLAQLSQNNINLLKNKHLLAFNQD**sv**YGQPA**tp**YK**w**GINPD**w**TFNVTYPAEFWAGPS**s**KGHLVLMVNTLDITATKEAKWNEIPGLSAGHYEVRDVWSDKDLGCLSSYKAAVAAHDTAVILVGKKCQRW

Score = 177 bits (450), Expect = 3e-47, Method: Compositional matrix adjust.

Identities = 130/384 (33%), Positives = 171/384 (44%), Gaps = 77/384 (20%)

Query: 2 **D**N**G**LA**R**TPTMGWLHWERFMCNLDCQEEPDSCISEKLFMEMAELMVSEGWKDAGYEYLCID 61

D + P++GW W + C++D E F+ AEL+VS G DAGY Y+ ID

Sbjct: 5 DGVTG**K**VPSLGWNSWNAYHCDID----------ESKFLSAAELIVSSGLLDAGYNYVNID 54

Query: 62 DCWMAPQRDSEGRLQADPQRFPHGIRQLANYVHSKGLKLGIYADVGNKTCAGFPGSFGYY 121

DCW +G + + RFP GI LA VH+ GLKLGIY+ G TCAG+P S GY

Sbjct: 55 DCWSMKDGRVDGHIAPNATRFPDGIDGLAKKVHALGLKLGIYSTAGTATCAGYPASLGYE 114

Query: 122 DIDAQTFADWGVDLLKFDGC--------------------------------------YC 143

D+DA FADWGVD LK+D C Y

Sbjct: 115 DVDAADFADWGVDYLKYDNCNVPSDWQDEYVACNPDFVKTGPNGTCTTALDPTLAPPGYD 174

Query: 144 DSLENLADGYKHMSLALNRTGRSIVYS-CEW---PLYMWPFQKPNYTEIRQYCNHWRNFA 199

S A+ + M AL + IV S C W ++ W N T I WR

Sbjct: 175 WSTSKSAERFGAMRNALAKQSHEIVLSMCIWGQADVFSW----GNSTGIS-----WRMSD 225

Query: 200 DIDDSWKSIKSILDWTSFNQERIVDVAG**P**GGWNDPDMLVIGNFGLSWNQQVTQMALWAIM 259

DI +W S+ IL+ SF VD G ND DML +GN L+ + T ALWA M

Sbjct: 226 DISPNWGSVTRILNLNS**F**K**LN**S-VD**F**WGH---NDADMLEVGNGNLTAAETRTHFALWAAM 281

Query: 260 AAPLFMSNDLRHISPQAKALLQDKDVIAINQDPL-GKQGYQLRQGDN----------FEV 308

+PL + DL +S LL++K ++A NQD + G+ + G N E

Sbjct: 282 KSPLLIGTDLAQLSQNNINLLKNKHLLAFNQD**SV**YGQPA**TP**YK**W**GINPD**W**TFNVTYPAEF 341

Query: 309 WERPLSGLAWAVAMINRQEIGGPR 332

W P S V M+N +I +

Sbjct: 342 WAGP-S**S**KGHLVLMVNTLDITATK 364

- **actual MIRs in query** (small red) = 04
- **MIRs in target** (small green)= 12
- **Common MIRs (True positive residues in query which were actually MIRs and also predicted as MIRs in alignment)** (highlighted in yellow) =01
